# Optineurin Cooperates With NRF2 to Regulate Tooth Root Morphogenesis by Controlling Mitochondrial Dynamics and Apoptosis

**DOI:** 10.1111/cpr.13799

**Published:** 2025-01-06

**Authors:** Haojie Liu, Xinyu Zhang, Xiao Ge, ChingCho Hsu, Yan Wang, Simai Chen, Xingzhi Yan, Rongyao Xu, Junqing Ma, Shuyu Guo

**Affiliations:** ^1^ Department of Orthodontics The Affiliated Stomatological Hospital of Nanjing Medical University Nanjing China; ^2^ State Key Laboratory Cultivation Base of Research Prevention and Treatment for Oral Diseases (Nanjing Medical University) Nanjing China; ^3^ Jiangsu Province Engineering Research Center of Stomatological Translational Medicine Nanjing China

**Keywords:** apoptosis, mitochondrial dynamics, nrf2, optn, tooth development

## Abstract

Tooth root development is a complex process essential for tooth function, yet the role of root dentin development in tooth morphogenesis is not fully understood. Optineurin (OPTN), linked to bone disorders like Paget's disease of bone (PDB), may affect tooth root development. In this study, we used single‐cell sequencing of embryonic day 16.5 (E16.5), postnatal day 1 (P1), and P7 mouse teeth, as well as embryonic and adult human teeth, to show that OPTN is vital for odontoblastic differentiation. In *Optn* ^−/−^ mice, we observed short root deformities and defective dentin, with impaired apical papilla differentiation and increased apoptosis. In vitro OPTN downregulation in stem cells of the apical papilla (SCAPs) exacerbated apoptosis and hindered odontoblastic differentiation. RNA‐seq analysis revealed significant differences in mitochondrial dynamics between control and OPTN knockout SCAPs. We discovered that OPTN influences mitochondrial dynamics primarily by promoting fission, leading to odontoblastic differentiation and mineralisation. Mechanistically, OPTN cooperates with NRF2 to regulate mitochondrial fission via DRP1 phosphorylation and affects the transcription of *BCL2*. Rescue experiments using an activator of NRF2 in ex vivo organ cultures and local gingival injection experiments confirmed these findings. Therefore, we concluded that OPTN, interacting with NRF2, acts as a key regulator of SCAPs mitochondrial dynamics, mineralisation and apoptosis during tooth development. These findings provide fresh insights into the mechanisms underlying tooth root development.

## Graphical abstract

OPTN interacts with NRF2 to facilitate the AKT/DRP1‐mediated regulation of mitochondrial fission in the cytoplasm of SCAPs, promoting tooth root mineralisation. Additionally, OPTN maintains NRF2's nuclear translocation, enhancing the transcriptional regulation of downstream BCL2, ultimately leading to the inhibition apoptosis in SCAPs and regulate tooth morphogenesis.

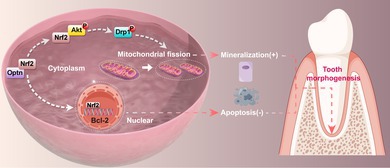

## 1 Introduction

Teeth are intricately specialised structures that perform vital roles in mastication and speech [1]. Their maintenance is critical for orofacial homeostasis and is intricately linked to systemic health and an individual's psychosocial well‐being [2]. The important process of tooth regeneration is to guide the development of tooth roots [3]. Tooth root development is a complex process that begins after crown formation. It involves the interaction between Hertwig's epithelial root sheath (HERS) of epithelial origin and the dental papilla derived from the ectomesenchyme [4]. The proliferation, apoptosis and differentiation of dental papilla stem cells, along with the regulatory influence of the HERS, are crucial for the formation of root dentin [5]. While various mutant animal models have demonstrated defects in root development, most studies have primarily documented these phenotypic defects without elucidating the underlying molecular regulatory network involved in root development.

OPTN serves as an autophagy receptor, playing a pivotal role in selective autophagy [6]. Mutations in the human OPTN gene have been identified in several familial diseases, including amyotrophic lateral sclerosis (ALS) and glaucoma [7, 8, 9, 10]. OPTN has been genetically linked to Paget's disease of bone (PDB), a chronic and disabling bone remodelling disorder often accompanied by severe orofacial deformities [11]. Reports have also revealed that patients with PDB exhibit abnormal tooth development, impaired dentin formation and shortened tooth roots [12, 13]. Recent research has highlighted OPTN as a pivotal molecular determinant in the cell fate determination of bone marrow mesenchymal stem cells (MSCs) [14]. This suggests that OPTN not only contributes to the regulation of orofacial bone development but also plays a crucial role in the regulation of tooth development. However, it remains unclear whether and how OPTN regulates tooth root development.

The tooth, as one of the major mineralised organs in the body, requires precise regulation of its mineralisation microenvironment during development [15]. As the major source of mineral granules in osteogenic cells, mitochondria are highly dynamic organelles, constantly undergoing fusion and fission, which significantly influence cellular functions. Mitochondrial dysfunction or imbalance can lead to impaired osteogenic differentiation and compromised bone formation [16]. However, the understanding of how mitochondrial components and processes, such as fission, specifically influence SCAPs and their odontogenic potential is still limited. Research indicates that the expression of OPTN E50K induces mitochondrial fission in glial lamina axons, suggesting its influence on mitochondrial dynamics [17]. How OPTN influences mitochondrial dynamics in tooth development is worth further investigation.

In the present study, single‐cell sequencing analysis suggests that OPTN may play an important role in odontoblast differentiation. Additionally, we generated genetically engineered mice and observed that while crown formation remained unaffected in the OPTN knockout mouse model, it led to delayed root elongation, ultimately resulting in shortened roots. Furthermore, we demonstrated that OPTN interacts closely with E2‐related factor 2 (NRF2), thereby governing apoptosis, mitochondrial dynamics and ultimately influencing odontoblastic differentiation in SCAPs.

## 2 Results

### 2.1 Spatiotemporal Expression Profiles of OPTN During Tooth Development

**FIGURE 1:**
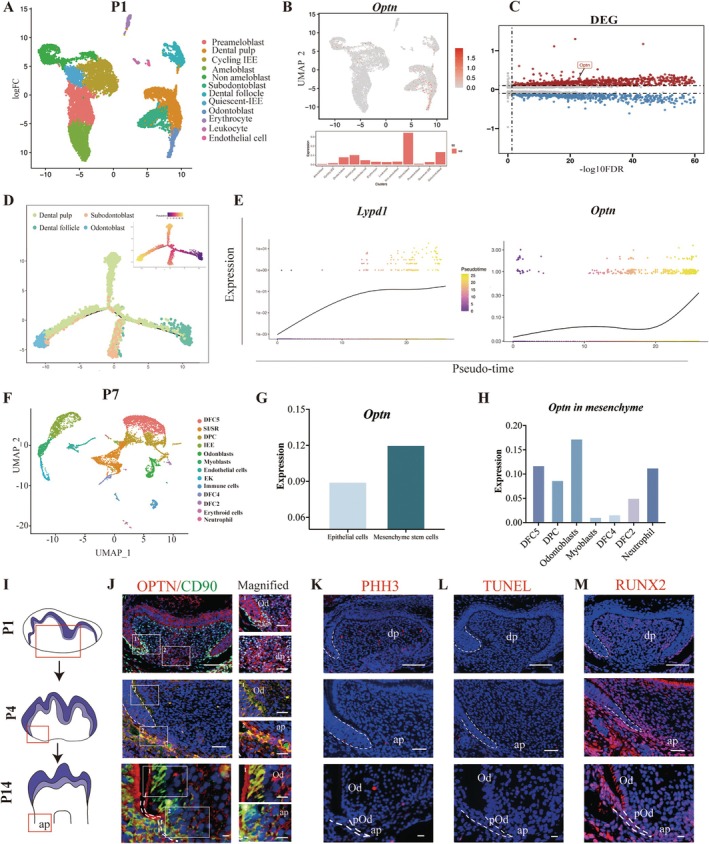
Expression pattern of OPTN in the developing tooth root. (A) The panel showed the results of cell population clustering based on known cell type marker genes at P1. (B) Expression of *Optn* was projected onto the UMAP plot (upper panel) and chart plot (lower panel). (C) Gene expression profile of P1 mesenchyme cells compared with that of the epithelial cells, according to different gene expression analyses. The highlighted plot represented *Optn*. (D) Trajectory analysis of dental mesenchymal subset including the dental pulp, odontoblasts, subodontoblasts and dental follicle cell populations coloured by cell types and pseudotime. (E) The graph showed the expression change of dental mesenchymal marker genes *Lypd1* and *Optn* according to pseudotime. (F) The panel showed the results of cell population clustering based on known cell type marker genes at P7. (G) Average gene expression of *Optn* between mesenchyme cells and epithelial cells. (H) Average gene expression of *Optn* in different cell clusters. (I) The schematic diagram of tooth development at P1, P4 and P14. (J) Representative images of immunofluorescent colocalisation of OPTN and CD90 in the developing tooth at P1, P4 and P14, respectively. Boxes in the left column images are enlarged in the right column images, representing the odontoblast (up) and apical region (down), respectively. The dotted lines indicate HERS. ap, apical papilla; dp, dental papilla; od, odontoblast; pOd, preodontoblast. Scale bars: 80 μm. (K) Representative images of the PHH3 staining, the TUNEL analysis (L) and the expression of RUNX2 with DAPI staining (M) in the developing tooth at P1, P4 and P14, respectively. Scale bars: 80 μm.

Tooth morphogenesis requires the mutual induction of epithelial and mesenchymal cells. During the period of tooth development, a wider variety of cell types emerge. Comprehending the cell varieties and the physiological processes that govern dental growth is crucial for elucidating the mechanism of tooth development. We analysed the scRNA‐seq datasets of E16.5, P1 and P7 mouse molars to understand the expression patterns of *Optn* gene at a single‐cell level. At E16.5, *Optn* was specifically expressed in dental papilla clusters 1 and 2, which highly express the odontoblast marker *Ibsp*, suggesting a potential role for *Optn* in odontoblast differentiation (Figure S1). At P1, cluster identification based on classic marker genes showed *Optn*'s enrichment within dental papilla mesenchyme, notably in odontoblasts and subodontoblasts (Figures 1A,B and S2C,D). Further differential gene expression analysis revealed *Optn* upregulation in mesenchyme compared to epithelial cells (Figures 1C and S2A,B). Trajectory analysis depicted increased OPTN and preodontoblast marker *Lypd1* at the late stage of differentiation, suggesting OPTN's role in odontoblastic differentiation and regulation in dental mesenchymal cells (Figure 1D,E). At P7, OPTN was prominent in mesenchyme and specifically detected in odontoblasts of mesenchyme origin (Figures 1F–H and S3). We further analysed the datasets from human embryonic molars (GSE184749) and adult human dental pulps (GSE164157) to examine the spatiotemporal expression of OPTN in human tissues. During the embryonic stage, OPTN showed significant average expression in the mesenchymal population of molars. In adults, OPTN had the highest average expression in the mesenchymal subpopulation among six subpopulations. These findings suggest an important role of OPTN in dental MSCs and validate conclusions from mouse single‐cell analyses (Figures S4 and S5).

To facilitate understanding of this intricate patterning process, we further investigated OPTN protein expression patterns at different tooth development stages (Figures 1I,J and S6). At E15.5, OPTN was widely distributed in the dental mesenchyme and epithelium. By P1, OPTN became more concentrated in the apical papilla and appeared in odontoblasts. As the root began to form at P4, OPTN was detectable in the apical papilla and odontoblasts. At P14, OPTN expression became prominent in the region of the apical papilla. The enriched apical expression of OPTN suggests that it may play a specific role in the mesenchyme and odontoblasts during tooth development. Additionally, OPTN co‐localised well with CD90 (a marker of the mesenchyme) in the dental mesenchyme during the embryonic stage and in the apical region during the postnatal stage. As we know, the proliferation, apoptosis and differentiation of dental papilla cells are crucial during root formation. Therefore, we detected the expression dynamics of these cell processes using PHH3, TUNEL and RUNX2 [18] staining (Figure 1K–M). The results confirmed that these functions are active in the apical region during root formation. However, the specific cell processes in dental papilla stem cells that OPTN influences require further investigation in subsequent studies.

### 2.2 OPTN‐Ablated Mice Exhibit Tooth Root Development Defects Accompanied by Apoptosis and Mineralisation Defects

**FIGURE 2:**
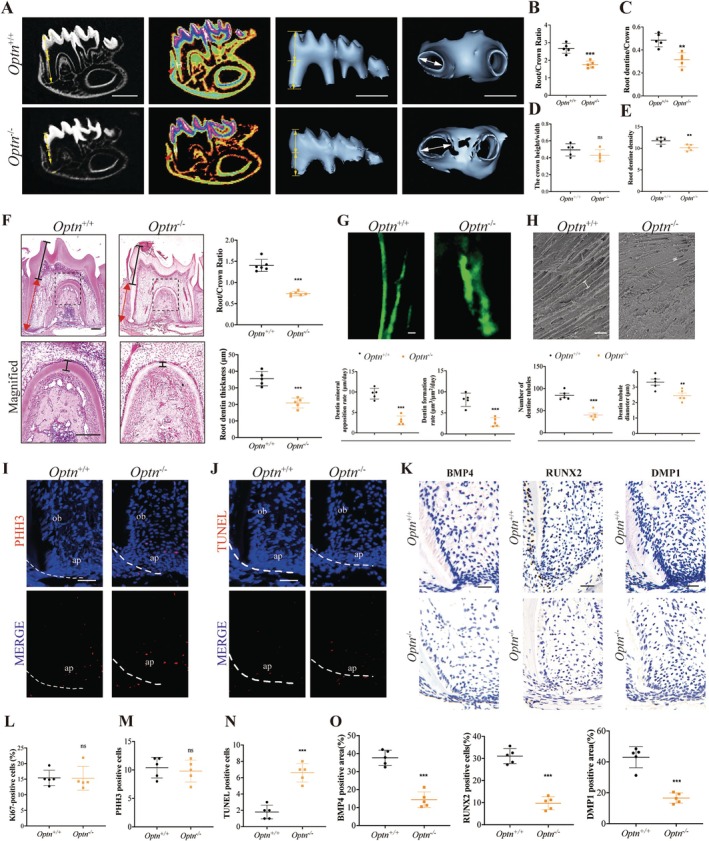
The loss of *Optn* resulted in root development defects in P21 mice. (A) Sagitta section and three‐dimensional reconstruction of mandibular dentition in the P21 *Optn* ^+/+^ and *Optn* ^−/−^ mice. Scale bars: 200 μm (left), Scale bars: 100 μm (right and middle). Double‐headed arrows (yellow) indicate tooth crowns and tooth roots. Double‐headed arrows (white) indicate the width of root apical regions. Red arrows indicate the thickness of the root dentine. Quantitative analysis of the root‐to‐crown ratio (B), root dentine‐to‐crown ratio (C), the crown height‐to‐width ratio (D) and root dentin density ratio (E). (F) H&E staining of tooth development in the *Optn* ^+/+^ and *Optn* ^−/−^ mice at P21. Scale bars: 30 μm. Dark lines indicate the length of the tooth crown, and red lines indicate the length of the tooth root (up). Dark lines in the lower pictures indicate root dentine. Boxes in the upper column images are enlarged in the down column images, respectively. Scale bars: 30 μm. Quantitative analysis of the root‐to‐crown ratio and root dentine thickness are on the right. (G) The representative of calcein double labelling (up) and quantification of mineral apposition rate and dentine formation rate in the dentine of *Optn* ^+/+^ and *Optn* ^−/−^ mice (below). Scale bars: 5 μm. (H) Photomicrographs of root dentin obtained by SEM (up), the quantification of the number of dentin tubules and dentin tubule diameters (below). White lines indicate dentin tubule diameters. Scale bars: 10 μm. (I) The PHH3 and (J) TUNEL analysis with DAPI staining in the apical regions of *Optn* ^+/+^ and *Optn* ^−/−^ mice at P21. Scale bars: 30 μm. The dotted lines indicate HERS. Ap, apical papilla; Ob, odontoblast. (K) Immunohistochemistry staining of apical regions showed the expression pattern of BMP4, RUNX2 and DMP1 in *Optn* ^+/+^ and *Optn* ^−/−^ mice at P21. Scale bars: 30 μm. (L) The quantitative analysis of Ki67 positive cell rate of root. (M, N) The quantitative analysis of (I, J). (O) Quantitative analysis of (K). The results are presented above as the mean ± SD. SD, standard deviation. *p < 0.05, **p < 0.01, ***p < 0.001, ns, no significance.

To explore the specific function of *Optn* in tooth development, we generated OPTN knockout mice (*Optn* ^−/−^ mice). The efficiency of OPTN knockdown has been verified (Figure S7B,C). Root development typically reaches completion around or after P18 [19]. Therefore, we investigated tooth development at P21. There was no obvious difference in the root number and crown morphology between control and *Optn* ^−/−^ mice molars. However, the ablation of *Optn* resulted in thinner apical dentin and shortened roots, while the length of the crowns remained largely unaffected (Figures 2A–C,F and S7D,E). Mico‐CT analysis of dentin density and crown height‐to‐width ratio showed that OPTN knockout led to a decrease in root dentin density without significantly affecting crown dentin density. Furthermore, OPTN knockout had no significant impact on the crown height‐to‐width ratio (Figures 2D,E and S7H). To exclude the possibility of delayed development in mutant mice, we extended our observations to P56. At this stage, similar defects in root length were consistently observed in the mutant mice (Figure S7A). The calcein staining results revealed a substantial decrease in the rate of dentine mineral apposition and formation in *Optn* ^−/−^ mice (Figure 2G). Besides, scanning electron microscopy (SEM) of *Optn* ^−/−^ mice displayed a reduction in both the number and diameter of dentine tubules when compared to *Optn* ^+/+^ mice (Figure 2H). These results indicated ablation of *Optn* disrupted the processes of dentinogenesis and root elongation, which is crucial for proper tooth development.

To determine whether the dental anomalies are caused by abnormal cell proliferation or cell death, we proceeded to assess cellular proliferation and apoptosis in the apical region of P21 mice. While Ki67 and PHH3 staining showed no obvious difference between *Optn* ^+/+^ and *Optn* ^−/−^ mice, TUNEL staining detected a notable increase in cell apoptosis in *Optn* ^−/−^ mice (Figures 2I–J,L–N and S7F–G,I). Dentinogenesis starts at bell‐stage of tooth development. Derived from dental papilla, odontoblasts are terminally differentiated and polarised odontogenic cells that synthesise and secrete dentin components. Therefore, we tested the odontoblastic differentiation level in mouse molar apical tissue using odontoblastic differentiation markers BMP4, RUNX2 [20] and DMP1 [21]. Results showed that the knockdown of *Optn* decreased the protein level of BMP4, RUNX2 and DMP1 (Figure 2K,O).

We further analyse whether OPTN affects the cell process of the crown. At E15.5, OPTN knockout did not significantly affect apoptosis in the dental mesenchyme (Figure S8A,B). However, at P4, OPTN knockout led to increased apoptosis in the root dental papilla, whereas there were no significant differences observed in the odontoblasts of the crown at the same time point (Figure S8C–E). At P21, proliferation (as measured by Ki67 staining), apoptosis (as measured by TUNEL assay) and differentiation (as indicated by DMP1 expression) in the crown odontoblasts were not affected by the OPTN knockout (Figure S8F–H). This suggests that OPTN may be required for normal apoptosis and odontoblastic differentiation of SCAPs during root development.

### 2.3 OPTN Deficiency Promotes Apoptosis and Inhibits Odontoblastic Differentiation In Vitro and Ex Vivo

**FIGURE 3:**
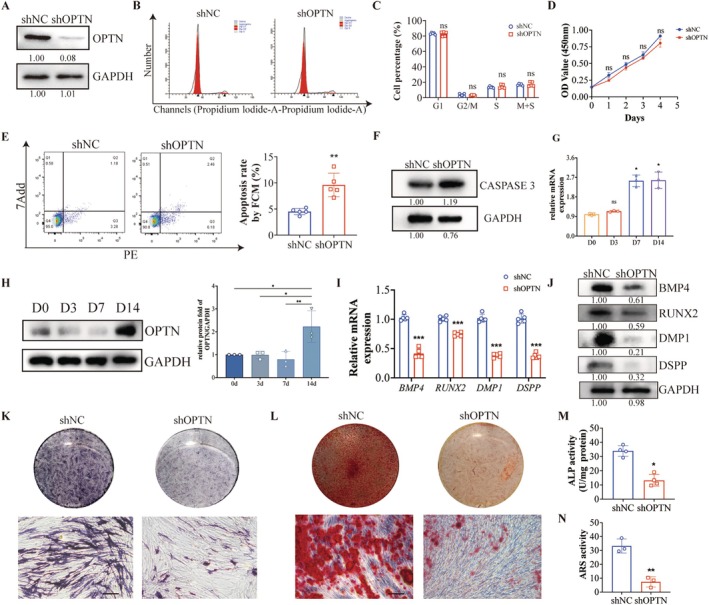
OPTN deficiency‐induced cell apoptosis to inhibit odontoblastic differentiation in SCAPs. (A) The WB shows OPTN knockdown efficiency. (B) Cell cycle profiles were generated by flow cytometry using PI staining with (C) relative statistical analysis on the right. (D) CCK8 analysis showed no significant differences in cell proliferation. (E) Cell apoptosis analysis and relative statistical analysis indicated an increased apoptosis rate in SCAPs infected with shOPTN lentivirus. (F) The protein level of apoptosis makers Caspase3 was detected by WB after mineralisation induction for 7 days with band quantification under the corresponding image. (G) The mRNA expression and (H) the protein level of OPTN in the process of mineralisation induction with relative quantification analysis on the right. (I) The mRNA level of odontoblastic genes such as *BMP4*, *RUNX2, DMP1* and *DSPP*. (J) The protein level of odonto/osteogenesis makers BMP4, RUNX2, DMP1 and DSPP was detected by WB after mineralisation induction for 7 days with band quantification under the corresponding image. (K) ALP staining and (L) ARS indicated SCAPs transfected with shOPTN lentivirus formed less calcified nodules than control. Scale bars: 80 μm. (M, N) quantitative analysis of ALP and ARS staining were on the right. The results are presented above as the mean ± SD. SD, standard deviation. *p < 0.05, **p < 0.01, ***p < 0.001, ns, no significance.

Then, we elucidate the function of OPTN in human SCAPs. Lentivirus was transfected to alter OPTN expression (Figure S9A). Stably transfected cells were categorised into shNC and shOPTN. Transfection efficiency was confirmed by WB (Figure 3A). The Cell Counting Kit‐8 (CCK‐8) assay and cell cycle analysis revealed no notable disparity in cell proliferation viability between the shNC and shOPTN groups (Figure 3B–D). Notably, we observed a discernible increase in the overall apoptosis rate (Q2 + Q3) in SCAPs transfected with shOPTN (Figure 3E). Additionally, the protein level of Caspase 3 also increased in the shOPTN group (Figure 3F). Besides, we used the apoptosis inhibitor Z‐VAD‐FMK (25 μM) in OPTN knockout SCAPs and stained them with TUNEL assay after 4 h to observe their apoptosis. The results showed that in OPTN knockout cells, the addition of Z‐VAD‐FMK reduced apoptotic SCAPs (Figure S9B,C). These outcomes signify that OPTN exerts a suppressive effect on apoptosis in SCAPs.

During tooth development, ensuring the proper differentiation and mineralisation of SCAPs is crucial for the development of healthy and functional teeth. To further study the OPTN function in the differentiation and maturation of SCAPs, we examined the expression of OPTN during the induction of odontogenic mineralisation in SCAPs. The expression of OPTN varies with mineralisation induction time. The mRNA level increases with the induction days and reaches its highest at 7 and 14 days. The protein expression levels were basically the same at 0 and 3 days, although the expression decreased at 7 days, it reached its peak again at 14 days (Figure 3G,H). Furthermore, mRNA and protein expression of odontoblastic markers DSPP, RUNX2, BMP4 and DMP1 were consistently downregulated upon OPTN inhibition (Figure 3I,J). Deletion of OPTN dramatically decreased alkaline phosphatase activity (ALP) activity and mineralisation nodule formation (Figure 3K–N). These results demonstrated that OPTN played a crucial role in odontoblastic differentiation. In summary, the *Optn* knockout led to an increase in apoptosis and hindered odontoblast differentiation.

### 2.4 OPTN Deficiency Inhibits Odontoblastic Differentiation by Restrained Mitochondrial Dynamics

**FIGURE 4:**
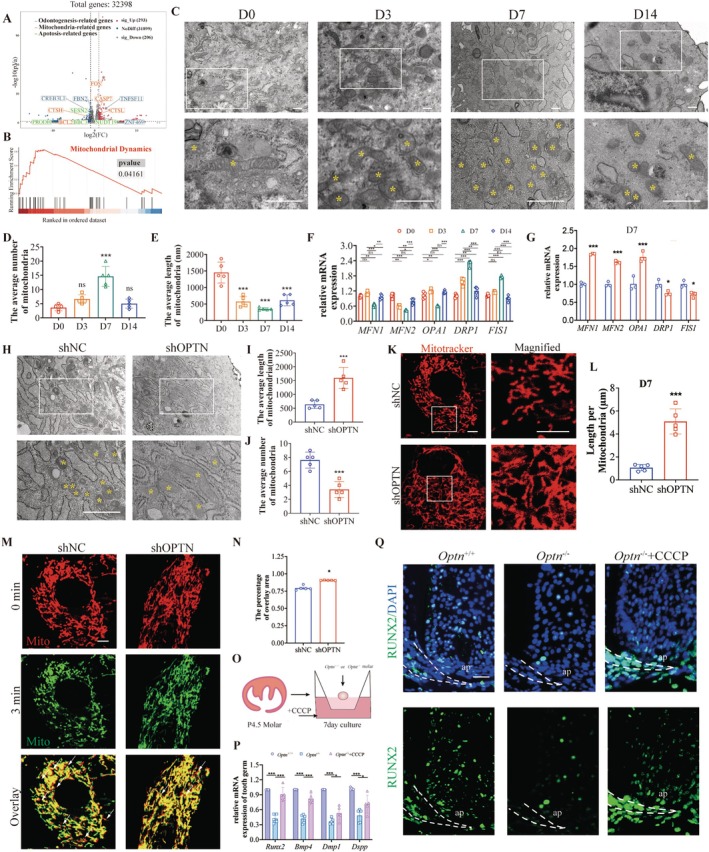
OPTN deficiency regulated mitochondria dynamics to inhibit odontoblastic differentiation in SCAPs. (A) The volcano plot of DEGs in SCAPs transfected with shNC or shOPTN lentivirus. (B) GSEA showed an enrichment of the gene set related to mitochondrial dynamics. (C) TEM images of mitochondria in SCAPs at differentiation days 0, 3, 7 and 14. Scale bars: 0.5 μm. Boxes in the first row are enlarged in the second row, respectively. Asterisks indicate mitochondria. Scale bars: 0.5 μm. (D) Quantitative analysis of mitochondria number and (E) mitochondria length with a total area of 50 μm^2^ in TEM image of mitochondria in SCAPs at differentiation days 0, 3, 7 and 14. (F) The mRNA level of mitochondrial fusion and fission‐related genes in SCAPs at differentiation days 0, 3, 7 and 14. (G) The mRNA level of mitochondria fusion and fission‐related genes in shNC and shOPTN SCAPs at differentiation day 7. (H) TEM image of shNC SCAPs and shOPTN SCAPs at differentiation day 7. Scale bars: 0.5 μm. Boxes in the first row are enlarged in the second row, respectively. Asterisks indicate mitochondria. Scale bars: 0.5 μm. (I) Quantitative analysis of mitochondria number and (J) mitochondria length of TEM image of shNC SCAPs and shOPTN SCAPs at differentiation day 7. (K) Representative images of mitochondria stained by mitotracker (red) in shNC and shOPTN SCAPs cultured in the mineralisation‐induced medium for 7 days. Scale bars: 10 μm. Boxes in the left column images are enlarged in the right column images, respectively. Scale bars: 10 μm. (L) Quantitative analysis of the length of mitochondria by mitotracker staining in (K). (M) SCAPs infected with shNC or shOPTN lentivirus were labelled with mitotracker Deep Red for live cell imaging. The overlay of the image at 0 min (pseudo colour of red) with the image at 3 min (pseudo colour of green) provides a visualisation of mitochondrial dynamics. Scale bar: 10 μm. Arrows indicate un‐overlapped areas. (N) Quantitative analysis of the mitochondrial dynamics in (M). (O) The schematic diagram of the ex vivo organ cultures with CCCP added to the culture medium for 7 days. (P) The mRNA expression of odontoblastic markers of *Optn* ^+/+^, *Optn* ^−/−^ and *Optn* ^−/−^ + CCCP tooth germs. (Q) Representative images of the expression of RUNX2 with DAPI staining in the mandibular first molar obtained from *Optn* ^+/+^, *Optn* ^−/−^ and *Optn* ^−/−^ + CCCP group with local gingival injection. Scale bar: 80 μm. Ap: Apical papilla. The dotted lines indicate HERS. The results are presented above as the mean ± SD. SD, Standard Deviation. *p < 0.05, **p < 0.01, ***p < 0.001. ns, no significance.

To investigate the reasons behind the increased apoptosis and impaired differentiation in OPTN knockout SCAPs, we performed RNA‐seq analysis. Data were obtained from SCAPs subjected to 7 days of mineralisation induction in both shNC and shOPTN groups (Table S2). Differentially expressed genes (DEGs) after OPTN knockdown were intersected with gene sets related to odontogenesis, apoptosis and mitochondrial function from Genecard, Mitocarta and MitoProteome. Notably, a significant proportion of DEGs was linked to these functions: odontogenesis (130 genes), mitochondria (33 genes) and apoptosis (5 genes) (Figure 4A). Further, Gene Set Enrichment Analysis (GSEA) revealed alterations in mitochondrial dynamics in the OPTN knockout group (Figure 4B). Mitochondrial components have been prominently identified in the bone matrix, and mitochondrial fission plays a significant role in regulating osteogenesis [22]; however, for SCAPs, its role remained unclear.

To validate the bioinformatics analysis results, we evaluated mitochondrial dynamics in both groups of SCAPs. First, we initially observed the changes in the mitochondrial network during the odontoblastic differentiation process (days 0, 3, 7, 14) by transmission electron microscopy (TEM) and quantitative reverse transcription PCR (qRT‐PCR). Results showed that the level of mitochondrial fragmentation gradually increased with the mineralisation day, reaching its peak at day 7, and then decreased again (Figure 4C–E). This pattern is consistent with our gene expression showing significant reductions in mitochondrial fusion marker genes (MFN1, MFN2, OPA1) and increases in mitochondrial fission marker (DRP1, FIS) upon odontogenic induction (Figure 4F). These results indicated odontogenic induction triggers mitochondrial fragmentation.

To investigate whether OPTN promotes odontoblastic differentiation via regulating mitochondrial dynamics, we explored changes of mitochondrial dynamics upon OPTN knockdown during differentiation days. TEM revealed an increase in the length of mitochondria and a decrease in their number upon OPTN knockdown, suggesting the inhibition of mitochondrial fission at day 7 (Figure 4H–J). A similar conclusion was drawn in Mitotracker staining (Figure 4K,L). Furthermore, the expression of mitochondrial dynamics marker genes verified that (Figure 4G). The observed less fragmented mitochondrial morphology in shOPTN SCAPs suggested a potential impairment in mitochondrial dynamics within SCAPs. To assess this, we employed live cell confocal imaging to record the dynamic movement of mitochondria in a time‐lapse 2D x‐y mode. For visualising mitochondrial dynamics in x‐y images, we selected the initial image at 0 s (pseudo colour of red) and the final image at 3 min (pseudo colour of green). These images were then superimposed to create an ‘overlay’ image. In the ‘overlay’, the yellow colour denoted a complete overlap of red and green, signifying no mitochondrial movement, predominantly observed in shOPTN SCAPs (Figure 4M,N). Conversely, red or green colours in the ‘overlay’ represented no overlap between mitochondria at these two moments, thus indicating mitochondrial movement during this period, more prominently in shNC SCAPs. Overall, OPTN knockdown hindered mitochondrial dynamics by impeding both fission and motility.

To address the impact of mitochondrial dynamics on odontoblastic differentiation, we utilised carbonyl cyanide m‐chlorophenyl hydrazine (CCCP), a mitochondrial uncoupler commonly employed to induce mitochondrial fission [23]. This investigation aimed to determine whether CCCP could rescue dentin formation by adding it to *an* ex vivo tooth germ culture medium or gingival injection of *Optn* ^−/−^ mice. Results showed a recovery in the mRNA levels of the odontoblastic differentiation marker (Figure 4O,P) in ex vivo *Optn* ^−/−^ tooth germ. In vivo, immunofluorescence staining showed that the number of RUNX2‐positive cells in the apical region increased in the *Optn* ^−/−^ + CCCP group (Figures 4Q and S12A).

We further analyse whether OPTN affects the mitochondrial dynamic of the crown. At E15.5 and P4, we observed that in *Optn* ^−/−^ mice, the cells in the apical papilla showed impaired mitochondrial fission. In contrast, the MSCs in the crown region did not show significant abnormalities in mitochondrial fission (Figure S10A–E). Therefore, we concluded that *Optn* knockout inhibited mitochondrial fission, thereby suppressing root odontogenic differentiation.

### 2.5 The Protein–Protein Interaction Between OPTN and NRF2 Affects Mitochondrial Fission and Cell Apoptosis

**FIGURE 5:**
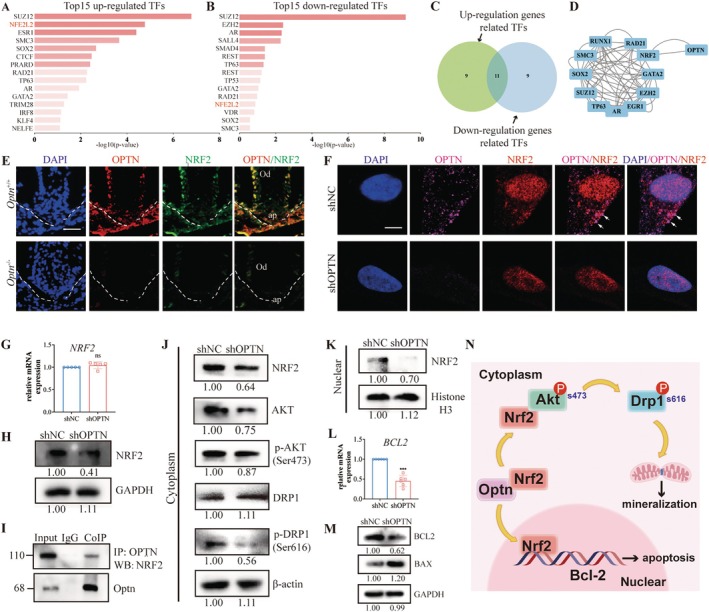
OPTN corresponded with NRF2 to activate mitochondrial fission and inhibit cell apoptosis. (A, B) The bar charts display the upstream regulatory transcription factors of upregulated and downregulated DEGs (The top 15 transcription factors are displayed). (C) The Venn diagram of co‐regulated transcription factors in (A, B). (D) The PPI networks between the predicted transcription factors and OPTN. (E) Representative images of immunofluorescent colocalisation of OPTN and NRF2 in P21 *Optn* ^+/+^ *and Optn* ^−/−^ mice. Arrowheads indicate the colocalisation zone. Scale bars: 100 μm. Dotted lines indicate the border between dental epithelium and dental mesenchyme. (F) Representative images of immunofluorescent colocalisation of OPTN and NRF2 in shNC SCAPs and shOPTN SCAPs. Arrowheads indicate the colocalisation of OPTN and NRF2. Scale bars: 10 μm. (G) qRT‐PCR showed no significant change of *NRF2* in shOPTN SCAPs. (H) WB showed relative protein levels of NRF2 in total cellular protein. (I) CO‐IP of OPTN with NRF2 in SCAPs extracts. (J) WB showed relative protein levels of NRF2, AKT, p‐AKT (Ser473), DRP1 and p‐DRP1 (Ser616) in cytoplasmic protein, and (K) WB showed relative protein levels of NRF2 in nuclear protein. (L) The mRNA level of *BCL2* of shNC and shOPTN SCAPs. (M) The total protein level of BCL2 and BAX of shNC and shOPTN SCAPs. (N) The schematic diagram of the regulation mechanism of mitochondrial fission and cell apoptosis in SCAPs during the development of dental root. The results are presented above as the mean ± SD. SD, standard deviation. *p < 0.05, **p < 0.01, ***p < 0.001, ns, no significance.

To elucidate the molecular mechanism by which OPTN affects downstream genes, we further analysed the RNA‐seq results. Next, we investigate the reasons for the changes in these downstream genes through RNA‐seq analysis. The X2k database was utilised to individually predict transcription factors for up‐ and downregulated DEGs (Figure 5A–C). Transcription factors within this intersection were then assessed for their potential intersection with OPTN through a protein–protein interaction (PPI) network (Figure 5D). Intriguingly, human NRF2 was identified. NRF2 is a transcription factor known for its role in cellular defence against oxidative stress [24]. NRF2 has been implicated in regulating the differentiation and function of odontoblasts [25]; however, the exact role of OPTN‐NRF2 in tooth development is still being investigated.

To answer this question, we visualised OPTN and NRF2 by immunostaining and confocal microscopy in mice tissue and SCAPs (Figure 5E,F). Colocalisation of OPTN and NRF2 was detected in the apical region of *Optn* ^+/+^ mice and the cytoplasm of shNC SCAPs. The NRF2 signal was decreased in both the nuclear and cytoplasmic when OPTN was knocked down. Then, the decreased level of NRF2 in total protein, without a concomitant change in mRNA levels upon OPTN knockdown, substantiates the results from immunofluorescence staining and implies that OPTN modulates NRF2 at the protein level (Figure 5G,H). Subsequently, a coimmunoprecipitation (CO‐IP) experiment confirmed the direct interaction between OPTN and NRF2 in SCAPs (Figures 5I and S11A). Western blot analysis further revealed that a reduction in OPTN expression resulted in decreased levels of NRF2 in the cytoplasm, accompanied by a reduced translocation of NRF2 into the nucleus (Figure 5J,K). These findings suggested that OPTN might interact with NRF2 to maintain NRF2 in the cytoplasm and its nuclear translocation in SCAPs.

A previous study reported that NRF2 activation caused activation of AKT and p‐AKT (S473) [26]. Moreover, activation of Ser kinase Akt‐triggered phosphorylation of DNM1L/DRP1 at 616 residues [27], which initiated mitochondrial fission. Based on this, our investigation extended to the examination of cytoplasmic protein levels, revealing that NRF2, AKT, p‐AKT and pDRP1 protein levels significantly decreased in the shOPTN group, as depicted in Figure 5J. We further treated OPTN‐ablated SCAPs with the pDRP1 inhibitor, Mdivi‐1, and performed ALP assays. We observed that the pDRP1 inhibition worsened the odontoblastic differentiation defect in OPTN‐ablated SCAPs (Figure S11B). Therefore, the results showed that the interaction between OPTN and NRF2 proteins may facilitate the AKT/DRP1‐mediated mitochondrial fission and further affect the differentiation of SCAPs.

B‐cell lymphoma 2 (BCL2) is a downstream target of NRF2 that plays a role in antiapoptosis [28]. Furthermore, we examined the expression of NRF2 in the nucleus and the expression of BCL2 in the whole cell. The results demonstrated a decrease in the nuclear protein level of NRF2, accompanied by reductions in both mRNA and total protein levels of BCL2 in the shOPTN group (Figure 5L,M). BCL2 is well known to inhibit BAX activity, thereby preventing mitochondrial membrane permeability and apoptosis [29]. WB analysis showed that the protein expression trend of BAX was opposite to that of BCL2 (Figure 5M). These findings suggest that the interaction between OPTN and NRF2 proteins plays a pivotal role in promoting the transcriptional regulation of downstream BCL2 by NRF2, ultimately leading to the inhibition of apoptosis in SCAPs (Figure 5N).

### 2.6 Activator of NRF2 Rescued Defects Caused by OPTN Deficiency in SCAPs and Mice Tooth

**FIGURE 6:**
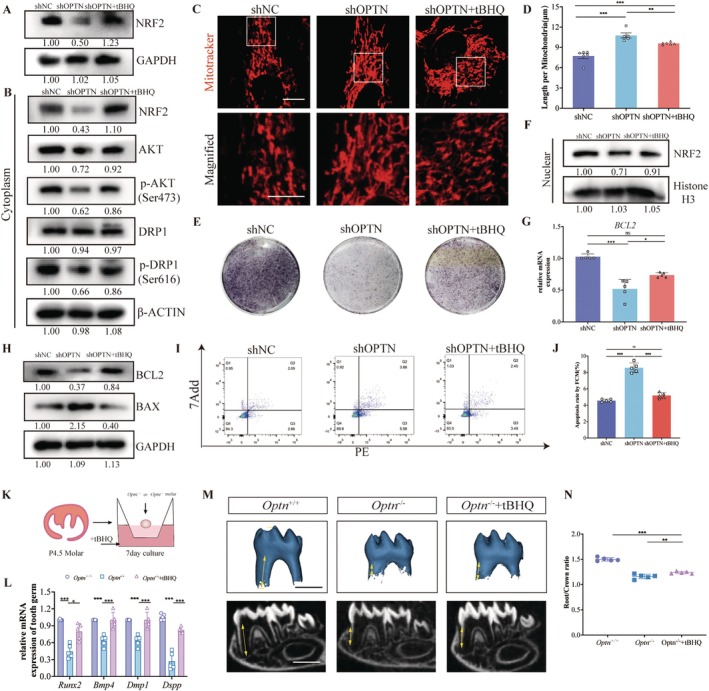
NRF2 activator partially rescued root development after knocking down OPTN. (A) The protein level of NRF2 in shNC, shOPTN and shOPTN + tBHQ SCAPs extracts. (B) Relative protein levels of NRF2, AKT, p‐AKT (Ser473), DRP1 and p‐DRP1 (Ser616) in cytoplasmic protein. (C) Representative images of shNC, shOPTN and shOPTN + tBHQ SCAPs cultured in mineralisation‐induced medium for 7 days stained with mitotracker. Scale bars: 10 μm. Boxes in the first row are enlarged in the second row, respectively. (D) The quantitative analysis of (C). (E) ALP staining of shNC, shOPTN and shOPTN + tBHQ SCAPs. (F) Relative protein levels of NRF2 in nuclear protein. (G) The mRNA of *BCL2* in shNC, shOPTN and shOPTN + tBHQ SCAPs. (H) The total protein level of *BCL2* and BAX in shNC, shOPTN and shOPTN + tBHQ SCAPs. (I) Cell apoptosis analysis and relative statistical analysis of shNC, shOPTN and shOPTN + tBHQ SCAPs with the quantitative analysis on the right (J). (K) The schematic diagram of the ex vivo organ cultures with tBHQ added to the culture medium. (L) The mRNA expression of odontoblastic markers of *Optn* ^+/+^, *Optn* ^−/−^ and *Optn* ^−/−^ + tBHQ tooth germs. (M) Three‐dimensional reconstruction and sagitta section of mandibular dentition in the *Optn* ^+/+^ mice group, *Optn* ^−/−^ mice group and *Optn* ^−/−^ + tBHQ group. Scale bars: 100 μm (up), Scale bars: 200 μm (down). Double‐headed arrows (yellow) indicate tooth roots. (N) Quantitative analysis of the root‐to‐crown ratio in (M). The results are presented above as the mean ± SD. SD, standard deviation. *p < 0.05, **p < 0.01, ***p < 0.001. ns, no significance.

Moreover, to further corroborate these in vitro findings and validate the influence mediated by the interaction between NRF2 and OPTN on tooth development, we applied an NRF2 activator, tert‐butylhydroquinone (tBHQ) [26], to SCAPs, ex vivo cultured tooth germ, and in vivo gingival injections. The group with tBHQ added is marked as shOPTN + tBHQ or *Optn* ^−/−^ + tBHQ. The activation effect of tBHQ on NRF2 has been verified (Figures 6A and S12B).

In the cytoplasm of SCAPs, the activation of NRF2 rescued the AKT/DRP1‐mediated regulation of mitochondrial fission and odontoblastic differentiation defect compared with the shOPTN group (Figure 6B–E). Furthermore, in the nucleus, the activation of NRF2 alleviated the transcription of BCL2, thereby reducing its protein level and reversing the BAX level (Figure 6F–H). Also, cell apoptosis in the shOPTN + tBHQ group decreased in comparison to the shOPTN group (Figure 6I,J).

**FIGURE 7:**
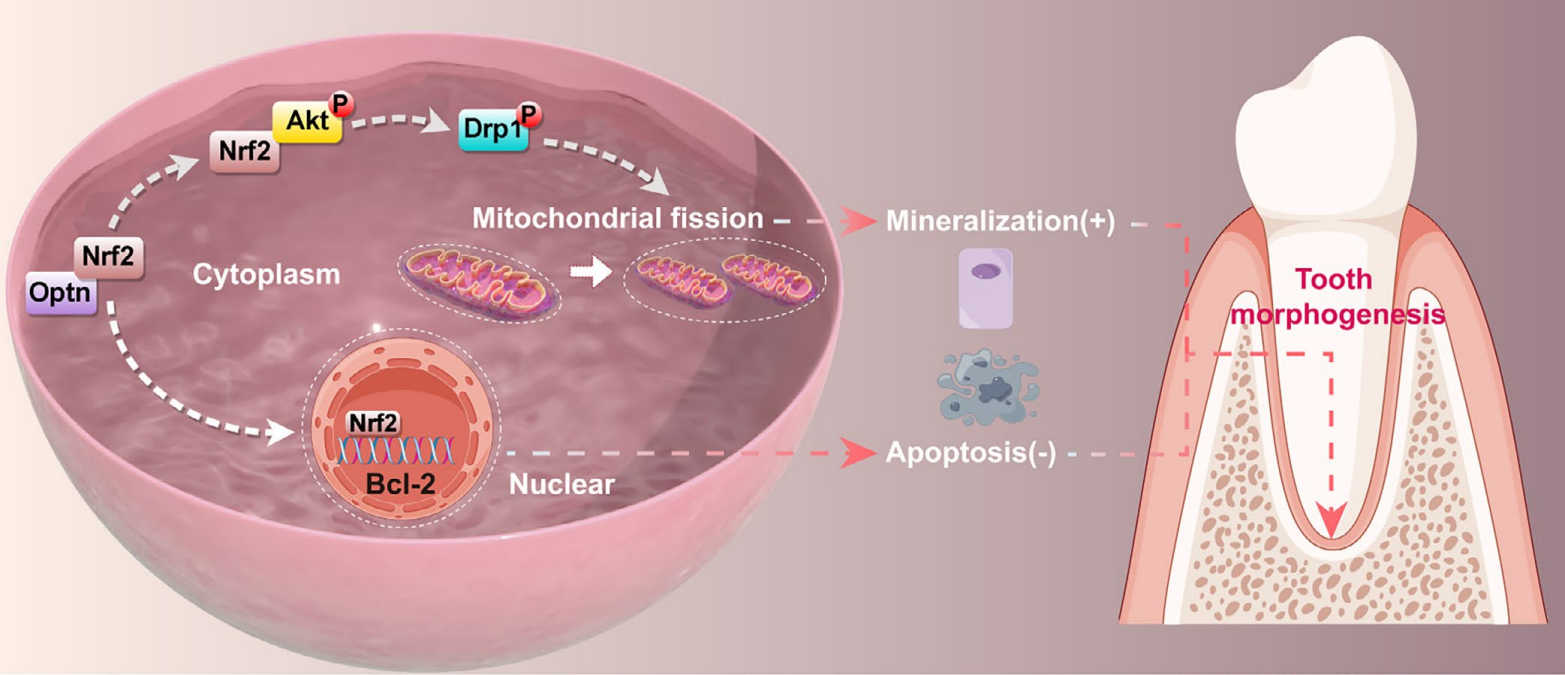
The schematic diagram of the regulation mechanism concerning OPTN interacting with NRF2 to regulate tooth morphogenesis by promoting mineralisation and inhibiting apoptosis. The figure was generated by Figdraw (www.figdraw.com).

Next, for tooth germs, we found that the mRNA levels of the odontoblast differentiation marker were rescued in the *Optn* ^−/−^ + tBHQ group compared with the *Optn* ^−/−^ group (Figure 6K,L). For the gingival injection rescue, the tooth length was significantly increased in the *Optn* ^−/−^ + tBHQ group compared with the *Optn* ^−/−^ group (Figure 6M,N). These results suggest that NRF2 is essential for odontoblastic differentiation in the developing teeth. These findings underscore the importance of NRF2 as an effective partner in collaboration with OPTN, playing a crucial role in the regulation of apoptosis and odontoblastic differentiation in SCAPs during the process of tooth root morphogenesis (Figure 7).

## 3 Discussion

The development of teeth and orofacial bones are intertwined as they follow common pathways during formation [30]. Mutations impacting genes pivotal in these pathways have the potential to disturb the typical development of both bones and teeth [21, 31]. Previous studies have documented OPTN as a crucial molecular factor in influencing the cell fate determination of BMSCs, playing a significant role in the regulation of orofacial bone development. However, the role of OPTN in tooth development has hitherto eluded understanding. Here, we have presented evidence indicating that OPTN plays a vital role in regulating odontogenesis and subsequently impacting root size during tooth development. Importantly, for the first time, we identified NRF2 as a potential collaborator of OPTN in root formation associated with mesenchymal cell mitochondrial dynamics and apoptosis during tooth morphogenesis.

Our findings showed that OPTN is expressed in SCAPs and odontoblasts of both the crown and root during tooth development. However, statistical comparison showed that *Optn* ^−/−^ mice displayed significant short root defects (root length, crown‐to‐root ratio, root dentin density), whereas alterations in dental crowns (crown height‐to‐width ratio, crown dentin density) were not notably apparent at the morphological level. This is likely attributable to the impairment of SCAP functions—such as apoptosis, mitochondrial fission and differentiation in the root, while the crown remains unaffected at the moleucular level. The molecular mechanisms underlying the formation of dental crowns and roots are distinct. Beyond combinatorial gene expression, root formation involves a more complex process that is influenced by factors such as HERS, adjacent anatomical structures and cranial neural crest (CNC)‐derived mesenchyme, which come into play after the completion of crown development [32, 33]. The regulation of OPTN and the activity of complex tissue–tissue interactions during root development warrant further investigation in future studies.

Recent research highlights the increasing attention on the significance of mitochondria in cellular function, particularly in the context of tooth development [16]. Mitochondrial quality control (MQC) is a comprehensive network that monitors the quality of mitochondria, serving as an endogenous protective mechanism crucial for maintaining mitochondrial homeostasis and functionality [34]. MQC coordinates various processes, including mitochondrial fission and fusion and mitophagy, to collectively regulate the maintenance of mitochondrial stability. Studies have explored the mechanism of OPTN as an autophagy receptor in mitophagy. However, research on the regulatory function of OPTN in mitochondrial fission and fusion, particularly in the context of tooth development, is notably limited. Here, we reveal that the knockdown of OPTN dramatically decreased the expression of odontoblastic markers. More importantly, we revealed that OPTN knockdown induced a change in mitochondrial shape, along with downregulated expression of DRP1, FIS, suggesting that OPTN promotes mitochondrial fission during odontoblastic differentiation. Considering the similarity of odontogenic and osteogenic mineralisation, our results are consistent with recent findings that increase mitochondrial fission through OPA1 downregulation during osteoblast maturation [22]. In this study, we identified OPTN as a novel downstream effector for activating mitochondrial fission in the context of odontoblastic differentiation, which updates molecular understandings about the role of OPTN in the maintenance of mitochondrial stability by orchestrating mitochondrial dynamics and mitophagy.

In light of the observed significant impact on mitochondrial fission and cell apoptosis in our study, we hypothesised that OPTN may collaborate with specific transcription factors to exert effects on downstream genes. Through RNA‐seq and bioinformatics analysis, we identified a potential candidate, NRF2. Previous research has demonstrated that OPTN can directly interact with both NRF2 [35] and its negative regulator, Kelch‐like ECH‐associated protein 1 (KEAP1) [36, 37]. This interaction between OPTN and NRF2 competitively reduces the binding of KEAP1 to NRF2, leading to NRF2 degradation via the ubiquitin‐proteasome system. This mechanism may explain the observed decrease in NRF2 protein levels with OPTN inhibition in SCAPs in our study.

In this study, using SCAP cells directly from *Optn* ^−/−^ mice would be more convincing than using human SCAP cells with shRNA‐mediated OPTN knockdown. However, the amount of apical papilla tissue in the molar teeth of developing mice is limited, making it difficult to obtain sufficient cells for large‐scale experiments. This issue is rarely addressed in the literature. While some studies have reported extracting apical papilla stem cells from mouse incisors [38, 39], these cells require immortalisation, which reduces their mineralisation capacity and does not align with the molar phenotype of interest. To ensure the reliability and validity of our results, we validated the effective knockdown of OPTN in human SCAP cells using Western Blot. Additionally, our in vivo data show that OPTN knockout leads to inhibited odontogenic differentiation and increased apoptosis in apical papilla cells, supporting our conclusions.

Formerly, tBHQ, a synthetic phenolic antioxidant widely used as a food additive, functions as a specialised activator of NRF2 [26]. This leads to reduced oxidative stress and enhanced antioxidant capacity by upregulating the NRF2 gene while suppressing nuclear factor kappa‐B (NF‐κB) activity [25]. In line with our findings demonstrating tBHQ's capacity to ameliorate short root anomalies in *Optn* ^−/−^ mice, a promising avenue emerges for potentially regenerating congenitally affected roots. Regenerative therapy may present an appealing option for patients with developmental tooth disorders. However, further research is essential to delineate the optimal drug delivery route and concentration for effective tooth development treatments.

## 4 Conclusion

In summary, OPTN plays a crucial role in tooth root development by affecting SCAPs' odontogenic differentiation and apoptosis. This occurs through its interaction with NRF2, which regulates mitochondrial fission via DRP1 phosphorylation in the cytoplasm while also influencing the transcription of the antiapoptotic BCL2 in the nucleus. The discovery of this OPTN/NRF2‐mediated mechanism provides new avenues for biomimetic mineralisation in tooth regeneration.

## 5 Experimental Section

### 5.1 ScRNA‐Seq Analysis

Data from the GSE167989 (P1), GSE162413 (E16.5 and P7), GSE184749 (human embryonic molars) and GSE164157 (adult human dental pulps) databases were processed using the R package Seurat 4.2. This encompassed foundational stages of cell normalisation and regression to obtain a representative and proportionate dataset. Considering potential batch effects, we applied the Harmony software package, with emphasis on the top 3000 variable genes. Additionally, for enhanced visualisation of cellular interactions and disparities, we employed UMAP technology for dimensionality reduction. Average expression function was used to quantify different gene expressions in different clusters. In human samples, we extracted cells, which OPTN expression is greater than 0. Then, average expression function was employed on these cells. To further clarify the cell types, we used the Findallmarker function combined with the R package SingleR and CellMarker website to identify different cell clusters in these data. We identified subodontoblast cluster with *Alpl*, odontoblast with *Bglap*, ameloblast with *Calb1*, cycling IEE with *Cks2*, dental follicle with *Col3a1*, quiescent IEE with *Foxp1*, endothelial Cell with *Gng1l*, erythrocyte with *Hbb‐bt*, preameloblast with *Igfbpl1*, nonameloblast with *Nectin4*, dental pulp with *Plac*. In bell‐stage germ, *Igfbp5* for Quiescent IEE, *Krt15* for erythrocyte, *Lgals7* for dental follicle, *Lyz2* for leukocyte, *Msx1* for mesenchymal linages, *Pecam1* for endothelial cell, *Pitx2* for epithelium linages and cycling IEE and *Postn* for dental papilla. In P7 tooth germ, *Cdkn2b* for IEE, *Hba‐a1* for erythroid cells, *Krt15* for SI/SR, *Lyz2* for immune cells, *Mgp* for myoblasts, *Pcp4* for DPC, *Pecam1* for endothelial cells, *Postn* for odontoblasts, *Shh* for EK and *Stfa2* for Neutrophil.

### 5.2 Trajectory Analysis

Given the dynamic nature of cellular development and differentiation processes, we employed the Monocle2 tool for single‐cell trajectory analysis. Preceding this, we identified crucial marker genes derived from Seurat's clustering outcomes and scrutinised expression counts within primitive cell populations. Leveraging the pseudotime trajectory, we subsequently applied the branch expression analysis model (BEAM analysis) to investigate pivotal nodes and regulatory genes orchestrating cell differentiation.

### 5.3 Gene Differential Expression Analysis

Gene differential expression analysis was conducted to gain a comprehensive understanding of cellular differentiation features. The Seurat package's FindCluster function was employed to partition cells, with specific attention directed toward discerning gene expression disparities between epithelial and mesenchymal cell populations. Stringent criteria were applied during the employment of the FindMarker function for differential expression analysis, necessitating a log_2_ fold change exceeding 0.15, a *p*‐value below 0.05 and a minimum percentage greater than 0.1, thereby ensuring that identified genes bear substantial biological relevance.

### 5.4 Gene Enrichment Analysis

To elucidate the functional implications of our analytical findings, we undertook gene enrichment analysis. Using the Org.Mm.eg.db as the reference database, we employed the ClusterProfiler function to probe into the functional categories and associated pathways of the identified genes. To provide a visual representation of these enrichment outcomes, we used the ggplot function for data visualisation.

### 5.5 Mice Breeding and Experiments

*Optn* ^+/−^ mice were procured from Shanghai Model Organisms, China. Specifically, we utilised a novel generated OPTN knockout mouse model targeting the Optn‐202 transcript (ENSMUST00000114996.7), with the aim of knocking out exon3. This project employed CRISPR/Cas9 technology to introduce mutations through nonhomologous recombination repair, resulting in frameshift and subsequent loss of function of the *Optn* gene. The process involved the following steps: obtaining Cas9 mRNA and gRNA through in vitro transcription; microinjecting Cas9 mRNA and gRNA into fertilised eggs of C57BL/6J mice to generate F0 generation mice; identifying positive F0 mice through PCR amplification and sequencing; mating positive F0 mice with C57BL/6J mice to obtain F1 mice. The F1 sequencing data are added to Table S3.

To generate *Optn* knockout mice, *Optn* ^+/−^ male mice were bred with *Optn* ^+/−^ females, and the genotype was verified through PCR, with the primer details provided in Table S1. The knockout mice constituted the *Optn* ^−/−^ group, while wild‐type mice from the same litters served as controls (*Optn* ^+/+^ group). All animal procedures were approved by the Ethics Committee of the Stomatological School of Nanjing Medical University and conducted following the guidance of the Animal Care Committee of Nanjing Medical University (IACUC‐2307024). A total of 60 mice from each group (*Optn* ^+/+^ and *Optn* ^−/−^) were raised until specific time points. The sole predetermined criteria for analysing each experiment were genotype and stage, without excluding any subjects. Subjects of both sexes were examined without distinction or exclusion, given that the studied gene, *Optn*, is autosomal. The mice were housed in clean, quarantined facilities with barriers, and measures were taken to minimise distress and pain throughout. At the designed experiment time point, anaesthesia and decapitation were performed. A precise dissection followed, involving the careful removal of skin and visceral tissues using scissors and forceps. Subsequently, mandibles and teeth were meticulously separated for further experiments, with a minimum of five mouse subjects examined per group.

### 5.6 Micro‐CT Analysis

Skulls from both *Optn* ^+/+^ and *Optn* ^−/−^ mice at postnatal day 21 (P21) and 56 (P56) were collected for CT scanning. Before scanning, specimens were fixed in 4% PFA overnight. Scanning was performed using a Micro‐CT system (vivaCT 80, Switzerland), with acquisition parameters set at 15.6 μm resolution, 50 kV voltage and 72 μA current. The ensuing CT image datasets were subjected to reconstruction and analytical scrutiny employing the CTvox v1.6, CTAn v1.13.8.1 and Mimics v21.0 software suites.

### 5.7 Immunohistochemical Staining and Immunofluorescence Staining

At various developmental time points, including embryonic day 15.5 (E15.5) and postnatal day 1 (P1), P4, P14 and P21, specimens from both *Optn* ^+/+^and *Optn* ^−/−^ mice were obtained. A 10% EDTA solution was used for specimen decalcification (21–40 days). Standard histological procedures were then followed, including dehydration, paraffin embedding and sectioning (5 μm slices). Subsequently, these tissue sections underwent immunohistochemical staining to elucidate any phenotypic variations and to evaluate the expression levels of odontogenesis‐related markers. Before staining, slides were subjected to serum blocking, followed by incubation with primary antibodies, including OPTN (sc‐166576, Santa Cruz, 1:200), BMP4 (ab39973, Abcam, 1:300), RUNX2 (98059s, Cell Signaling Technology, 1:300), DRP1 (sc271583, Santa Cruz, 1:200), TOM20 (D8T4N, Cell Signaling Technology, 1:300), DMP1 (bs‐12359R, Bioss, 1:300), NRF2 (16396‐1‐AP, Proteintech, 1:200), Ki67(ab16667, Abcam, 1:200), CD63 (ab217345, Abcam, 1:200) and PHH3 (sc‐8656‐R, Santa Cruz, 1:50) as markers of cellular proliferation. On the subsequent day, slides were subsequently incubated with immunohistochemical secondary or fluorescence‐conjugated secondary antibodies as appropriate, with counterstaining accomplished through the use of either DAB (DAB‐0031, MXB, Fujian, China) or DAPI (C1005, Beyotime, Jiangsu, China).

### 5.8 Calcein Double Labelling

Mice received intraperitoneal injections of the first dose of calcein (30 mg/kg, Sigma‐Aldrich) 17 days before euthanasia. Second calcein injections were given 3 days before euthanasia. At the end of the study, all animals were anaesthetised, sacrificed and dehydrated using ethanol series (70%, 80%, 90%, 100%) and infiltrated with Technovit 7200 resin (Heraeus KULZER, Hanau, Germany) for 2 weeks. After being embedded in methyl methacrylate, 10 μm longitudinal sections were examined under fluorescent microscopy to evaluate the mineral apposition rate (MAR) and dentine formation rate (DFR) following the calculation method as described previously [40].

### 5.9 SEM Analysis

SEM was used to observe the surface morphologies of the samples after treatment. The samples were sputter‐coated with gold and examined using SEM (JEOL JSM‐7900F, Tokyo, Japan) using conditions of 3 kV at magnification ranging from 500× to 2000×. Mandible with the tooth from both *Optn* ^+/+^ and *Optn* ^−/−^ mice at postnatal day 21 (P21) were fixed in 2.5% glutaraldehyde at 4°C no longer than a month before their use. Dentine discs with a thickness of 2–3 mm were sectioned along the coronal plane of the mandible on the distal surface of the first molar using dental burs. The specimens were processed in 37% phosphoric acid gel etch for 30 s, followed by rinsing with distilled water (DW) for 5 min, and then were kept in 2.5% glutaraldehyde at 4°C until observing. The number of dentin tubules from photomicrographs with a total area of 3000 μm^2^, counting all present and unobstructed tubules in each root surface. Only tubules that showed an almost circular lumen were selected to calculate diameters.

### 5.10 Cell Culture

Developing apical papilla tissues were obtained from healthy donors (18–25 years old) immature third molars at the Jiangsu Provincial Stomatological Hospital with ethical approval. SCAPs were extracted from the apical papilla tissues following previously described methods [41, 42]. The quality control and technical specifications for SCAPs comply with the guidelines outlined in ‘Human Mesenchymal Stem Cells’ [43]. The cultivation of these cells was executed in α‐minimum essential medium (α‐MEM), following established protocols [44]. For the rescue experiments, 50 μM of tBHQ (HY‐100489, #MCE, USA) was added to cells for 96 h post lentiviral transfection. Experiments utilised cells from passages 3 to 5.

### 5.11 Lentiviral Transfection

One day preceding lentiviral infection, stem cells from SCAPs were plated in large culture dishes at a density of 1 × 10^6^ cells per dish and allowed to culture overnight. When cell confluence reached approximately 70%, the culture media were substituted with a medium devoid of penicillin–streptomycin, supplemented with 5 μg/mL polybrene. Concurrently, lentiviral particles carrying shRNA sequences targeting the OPTN gene (shOPTN 5′‐GGGCTCAGATGGAAGTTTA‐3′) or a negative control shRNA (shNC 5′‐TTCTCCGAACGTGTCACGT‐3′) were introduced at a multiplicity of infection (MOI) of 30. After 24 h, the transfection medium was changed, and the transfection efficiency was checked under the microscope. RNA was extracted at 48 h, and protein was extracted at 72 h to check OPTN knockdown efficiency.

### 5.12 Cell Proliferation Assay

To assess the impact of OPTN knockdown on the proliferation of SCAPs, the CCK‐8 (Dojindo Kagaku Co, Kumamoto, Japan) was used. SCAPs transfected with either shNC or shOPTN lentivirus were seeded at 8 × 10^3^ cells per well in 96‐well plates and incubated at 5% CO_2_ 37°C for 12 h (day 0). At various time intervals (specifically, days 0, 1, 2, 3 and 4), the CCK‐8 reagent, mixed with fresh culture medium at a 1:10 ratio, was introduced into each well. After 1 h of incubation, optical density (OD) was detected at 450 nm using a microplate reader (Spectramax190, MD, Japan).

### 5.13 Flow Cytometry Assay

Flow cytometry assessed cell cycle progression and apoptosis in SCAPs after shNC or shOPTN transfection. Cell cycle analysis used PI staining, while apoptosis was evaluated with Annexin‐V‐PE and 7AAD. Analysis of the samples was conducted via FACS can cytometry (Becton‐Dickinson, SanJose, CA, USA). Subsequent data analysis was carried out using Flowjo V7 software.

### 5.14 Western Blotting

Total cellular proteins were harvested following the previously delineated protocol [45]. Cytoplasmic and nuclear fractions were isolated using an NE‐PER kit. Equal protein amounts were loaded onto 10% or 12% SDS‐PAGE gels and transferred to PVDF membranes. After blocking, membranes were probed overnight at 4°C with primary antibodies against OPTN (ab213556, Abcam, 1:1000), BMP4 (ab39973, Abcam, 1:1000), RUNX2 (98059s, Cell Signaling Technology, 1:1000), DSPP (sc73632, Santa Cruz, 1:800), DMP1 (bs‐12359R, Bioss, 1:1000), NRF2 (16396‐1‐AP, Proteintech, 1:2000) pDRP1‐S616 (AF8470, Affinity, 1:1000), p‐AKT‐S473 (AF0016, Affinity, 1:1000), DRP1 (sc271583, Santa Cruz, 1:800), beta Actin (AF7018, Affinity, 1:2000), Histone H3 (4499S, Cell Signaling Technology, 1:2000), BAX (sc65532, Santa Cruz, 1:1000), Caspase 3 (sc7272, Santa Cruz, 1:800) or GAPDH (MB001, Bioworlde, 1:2000). The subsequent day, the membranes underwent incubation with a secondary antibody (1:8000, KPL, USA), washed and exposed. ImageJ software was used for semi‐quantitative analysis.

### 5.15 RNA‐Seq

TRIzol reagent (Invitrogen, USA) was used for RNA extraction. For RNA‐seq, RNA samples of SCAPs transfected with shNC or shOPTN following a 7‐day incubation period in the osteogenic medium were sequenced by Lianchuan (Hangzhou, China) and analysed using the OmicStudio tools at https://www.omicstudio.cn/tool. Differential expression was estimated by selecting transcripts that changed with a significance of *p* < 0.05, |log_2_fold change| > 0.5849 and FDR < 0.05. Expression2Kinases (X2K) (X2K Web: https://maayanlab.cloud/X2K/) [46] was utilised to investigate the upstream regulatory networks from the DEGs signatures in SCAPs transfected with shNC or shOPTN. The transcriptional profiles were listed in Table S2.

### 5.16 Transmission Electron Microscopy

SCAPs in a mineralisation medium were fixed in 2.5% glutaraldehyde, treated with 1% osmium tetroxide, dehydrated and embedded in epoxy resin. Thin sections (about 90 nm) were placed on carbon‐coated nickel grids and examined using a JEM‐1400 Flash (JEOL, Tokyo, Japan).

### 5.17 Active Mitochondria Staining

SCAPs cultured in mineralisation medium for 7 days were stained with 100 nM MitoTrackerTM Red FM (Beyotime, China) for 5 min at 37°C. Confocal Laser Scanning Microscopy (CarlZeiss LSM710, Germany) was used to examine mitochondrial morphology and motility. Live cell imaging settings involved capturing one picture at 50‐s intervals for 3 min. The quantification of mitochondrial network parameters in each group was performed using an ImageJ‐based tool. Additionally, the calculation of the overlay area of the first and the last live cell images was utilised to evaluate mitochondrial motility.

### 5.18 Co‐Immunoprecipitation

Co‐IP was conducted using the Protein A/G Magnetic Beads IP Kit following the manufacturer's guidelines. The targeted antibody (2 μg) and normal mouse IgG (sc‐2025, Santa Cruz, USA) were diluted in 500 μL of PBST. Subsequently, 20 μL of Protein A/G magnetic beads (P2108, Beyotime, Jiangsu, China) were added to the mixture, and the incubation was carried out for 8 h at 4°C. After removing the supernatant using a magnetic separator, antibody‐conjugated immunomagnetic beads were prepared. Following the harvesting, lysis and centrifugation of protein samples, the supernatants were gently mixed with antibody‐conjugated immunomagnetic beads for an additional 8 h at 4°C to create an immunomagnetic beads‐antibody‐antigen complex. After washing the beads with PBST three times, the complex was resuspended in 60 μL of PBST and utilised for detecting endogenous interactions between the targeted antibody and other proteins through western blotting.

### 5.19 Quantitative Reverse Transcription PCR for mRNA Analysis

Total cellular RNA was extracted employing an RNA isolation kit (Vazyme, Nanjing, China). Complementary deoxyribonucleic acid (cDNA) was synthesised using 5× HiScript II qRT supermix (Vazyme, Nanjing, China). Subsequently, qRT‐PCR reactions were conducted employing 2X Taq Pro Universal SYBR qPCR Master Mix (Vazyme, Nanjing, China) on the ABI‐7200 Real‐Time PCR System (Applied Biosystems, CA, USA). The specific primer sequences utilised are detailed in Table S1 below. Data analysis was carried out utilising the $2^{-\Delta \Delta C_{T}}$ method.

### 5.20 ALP Assay and Alizarin Red Staining

Following a 7‐ or 14‐day incubation period in an osteogenic medium. The osteogenic induction medium was composed of α‐MEM, supplemented with 10% FBS, 50 μg/mL L‐ascorbic acid 2‐phosphate, 10 nM dexamethasone, 1.8 mM potassium dihydrogen phosphate and 10 mM β‐glycerophosphate. SCAPs were fixed using a 4% paraformaldehyde (PFA) solution and subsequently stained using the BCIP/NBT Alkaline Phosphatase Colour Kit (Beyotime, Jiangsu, China) or 2% Alizarin Red solution (ARS; Sbjbio, China). To measure ALP activity, an ALP activity kit (Beyotime, Shanghai, China) was employed, with values normalised to protein concentrations. Mdivi‐1 (MCE, HY‐15886, 10 μM) was added into the medium at the beginning for 48 h to inhibit p‐Drp1 (Ser616) level in the shOPTN + Mdivi‐1 group [47]. Following this, the ALP assay was conducted.

### 5.21 TUNEL Assay

Sections (5 μm) from P21 *Optn* ^+/+^and *Optn* ^−/−^ mice mandible were deparaffinised, rehydrated and washed with PBS. They were treated with 0.5% Triton X‐100, followed by immersion in TUNEL Label and Enzyme solutions (Roche Applied Science (Penzberg, Germany)) at 37°C for 1 h. Subsequently, sections were incubated with DAPI for 5 min at room temperature. For the TUNEL assay in SCAPs, we used the apoptosis inhibitor Z‐VAD‐FMK (25 μM) [48] in OPTN knockout SCAPs and stained them with TUNEL after 4 h to observe apoptosis.

### 5.22 Ex Vivo Organ Cultures

Tooth germs from P4 *Optn* ^+/+^and *Optn* ^−/−^ mice molars were cultured in cell culture inserts (labselect, China), which allowed free substance exchange between inserts and 24 well plates using an air–liquid interface technique for 7 days, as described [49]. The grouping is as follows: *Optn* ^+/+^ mouse tooth germ with DMSO, *Optn* ^−/−^ mouse tooth germ with DMSO, *Optn* ^−/−^ mouse tooth germ with tBHQ or CCCP. tBHQ was added to the culture medium of the rescue group at a dose of 50 mg/kg. CCCP was added to the culture medium of the rescue group at a dose of 40 mg/kg. In contrast, 0.5% of DMSO served as a vehicle control. Tooth germs were harvested after 7 days to assess the odontoblast differentiation with qRT‐PCR.

### 5.23 In Vivo Gingival Injection

We performed in vivo validation through periodontal injection. We followed the established method of local drug injection into the mouse gingiva [50, 51, 52], optimising the procedure and dosage according to the in vivo concentrations of CCCP and tBHQ. Specifically, From the initial injection on P14, injections were given daily for 7 consecutive days. Approximately 6 μL was injected into the gingival margin above the first molar on both sides of the lower jaw using a 10‐μL Hamilton syringe (World Precision Instruments, Sarasota, FL). The rescue group received tBHQ (1.245 mg/kg) or CCCP (1.5 mg/kg) in *Optn* ^−/−^ mice, while the control group received 1% DMSO. Mice were euthanised on P21, and samples were collected. We further extracted proteins from the root and surrounding tissues of the first molar and conducted WB experiments to verify the success of modelling and the effectiveness of the drug.

### 5.24 Statistical Analysis

The statistical details are outlined in the figure legends. GraphPad Prism was employed for statistical analysis, and significance was assessed using either the Student's *t*‐test or one‐way ANOVA test. The *p*‐values ≤ 0.05 (*), ≤ 0.01 (**) and ≤ 0.001 (***) were deemed statistically significant. All experiments were repeated no less than three times (*n* ≥ 3); ‘*n*’ represents independent values, not replicates.

## Author Contributions

Haojie Liu, contributed to the conception, design, data acquisition and analysis, drafted and critically revised the manuscript; Xinyu Zhang, contributed to data acquisition and statistical analysis, and drafted the manuscript; Xiao Ge, contributed to the bioinformatic analysis; ChingCho Hsu, contributed to CT data analysis; Yan Wang, contributed to sketch drawing; Simai Chen, contributed to sketch drawing; Xingzhi Yan, contributed to data processing; Rongyao Xu, contributed to the conception, design; Junqing Ma, contributed to the conception, design, drafted and critically revised the manuscript; Shuyu Guo, contributed to the conception, design, drafted and critically revised the manuscript. All authors gave their final approval and agreed to be accountable for all aspects of the work.

## Conflicts of Interest

The authors declare no conflicts of interest.

## Supporting information


Data S1

## Data Availability

All original data can be obtained from the authors upon reasonable request.
