# Supplementary material for: Optineurin Cooperates With NRF2 to Regulate Tooth Root Morphogenesis by Controlling Mitochondrial Dynamics and Apoptosis
Source: Cell Prolif. 2025 Jan 6;58(5):e13799. doi: 10.1111/cpr.13799 (PMC12099217; doi:10.1111/cpr.13799)
Supplement: Supplementary file 1 — Data S1 [file CPR-58-e13799-s001.pdf]

Supplementary Materials for

Optineurin cooperates with NRF2 to regulate tooth root morphogenesis by controlling mitochondrial dynamics and apoptosis

Haojie Liu[1, 2, 3*], Xinyu Zhang[1, 2, 3*], Xiao Ge[1, 2, 3*], ChingCho Hsu[1], Yan Wang[1, 2, 3], Simai Chen[1, 2, 3], Xingzhi Yan[1, 2, 3], Rongyao Xu[1, 2, 3], Junqing Ma[1, 2, 3] ✉ & Shuyu Guo[1, 2, 3] ✉

[1]Department of Orthodontics, The Affiliated Stomatological Hospital of Nanjing Medical University, Nanjing, China.

[2]State Key Laboratory Cultivation Base of Research, Prevention and Treatment for Oral Diseases (Nanjing Medical University), 140 Hanzhong Road, Nanjing 210029, China.

[3]Jiangsu Province Engineering Research Center of Stomatological Translational Medicine, Nanjing, Jiangsu Province, 210029, China.

[*]These authors contributed equally to this work.

Correspondence authors:

S. Guo, Department of Orthodontics, the Affiliated Stomatological Hospital of Nanjing Medical University, 140 Hanzhong Road, Nanjing 210029, China. E-mail: syguo@njmu.edu.cn.

J. Ma, Department of Orthodontics, the Affiliated Stomatological Hospital of Nanjing Medical University, 140 Hanzhong Road, Nanjing 210029, China. E-mail: jma@njmu.edu.cn

This file includes:

Figure S1 to S12

Table S1 to S3

Table S1. Primers used in this study

Gene	Forward	Reverse
**mus musculus (PCR)-*Optn* (1)**	CTTTGTTTCAGGGGAGGGGT	TCCAGATAGCAGCAAGGAGCTT
**mus musculus (PCR)-*Optn* (2)**	TTATTTATTTGCTGACATACCCATCC	CATCCTAATCTCTGCCCATCTACTCT
**mus musculus-*Gapdh***	ACCACAGTCCATGCCATCAC	TCCACCACCCTGTTGCTGTA
**mus musculus-*Dmp1***	CTGAAGAGAGGACGGGTGATT	CGTGTGGTCACTATTTGCCTG
**mus musculus-*Dspp***	ATTCCGGTTCCCCAGTTAGTA	GCCTTCCTCTATCACCTTC
**mus musculus-Runx2**	AGAGTCAGATTACAGATCCCAGG	TGGCTCTTCTTACTGAGAGAGG
**mus musculus-Bmp4**	TTGATACCTGAGACCGGGAAG	GGCGACTCAGAGCACAAG
**mus musculus-*Drp1***	CAGGAATTGTTACGGTTCCCTAA	CCTGAATTAACTTGTCCCGTGA
**mus musculus-*Opa1***	TGGAAAATGGTTCGAGAGTCAG	CATTCCGTCTCTAGGTTAAAGCG
**mus musculus-*Mfn1***	ATGGCAGAAACGGTATCTCCA	GCCCTCAGTAACAAACTCCAGT
**mus musculus-*Fis1***	AGAGCACGCAATTTGAATATGCC	TGTGCCCCACGCCCTGATTC
**mus musculus-*Mfn2***	AGAACTGGACCCGGTTACCA	CACTTCGCTGATACCCCTGA
**homo sapiens-*GAPDH***	GAAGGTGAAGGTCGGAGTC	GAGATGGTGATGGGATTTO
**homo sapiens-*FIS1***	TGGTGCGGAGCAAGTACAAT	TGCCCACGAGTCCATCTTTC
**homo sapiens-*BMP4***	CTCCAAGAATGGAGGAGCTGTAGGAA	CCTATGAGAGAGCAGGCAAGA
**homo sapiens-*DMP1***	GGAAGAGGTGGTGAGTGAG	TTGAGTGGGAGAGTGTG
**homo sapiens-*DSPP***	CCATTCCAGTTCCTCAAA	GCCTTCCTCTATCACCTTC
**homo sapiens-*RUNX2***	AGTTCCCAAGCATTTCATC	GGCAGGTAGGTGTGGTAGT
**homo sapiens-*DRP1***	CTGCCTCAAATCGTCGTAGTG	GAGGTCTCCGGGTGACAATTC
**homo sapiens-*OPA1***	TGTGAGGTCTGCCAGTCTTTA	TGTCCTTAATTGGGGTCGTTG
**homo sapiens-*MFN1***	GTTGCCGGGTGATAGTTGGA	TGCCACCTTCATGTGTCTCC
**homo sapiens-*MFN2***	CTCTCGATGCAACTCTATCGTC	TCCTGTACGTGTCTTCAAGGAA

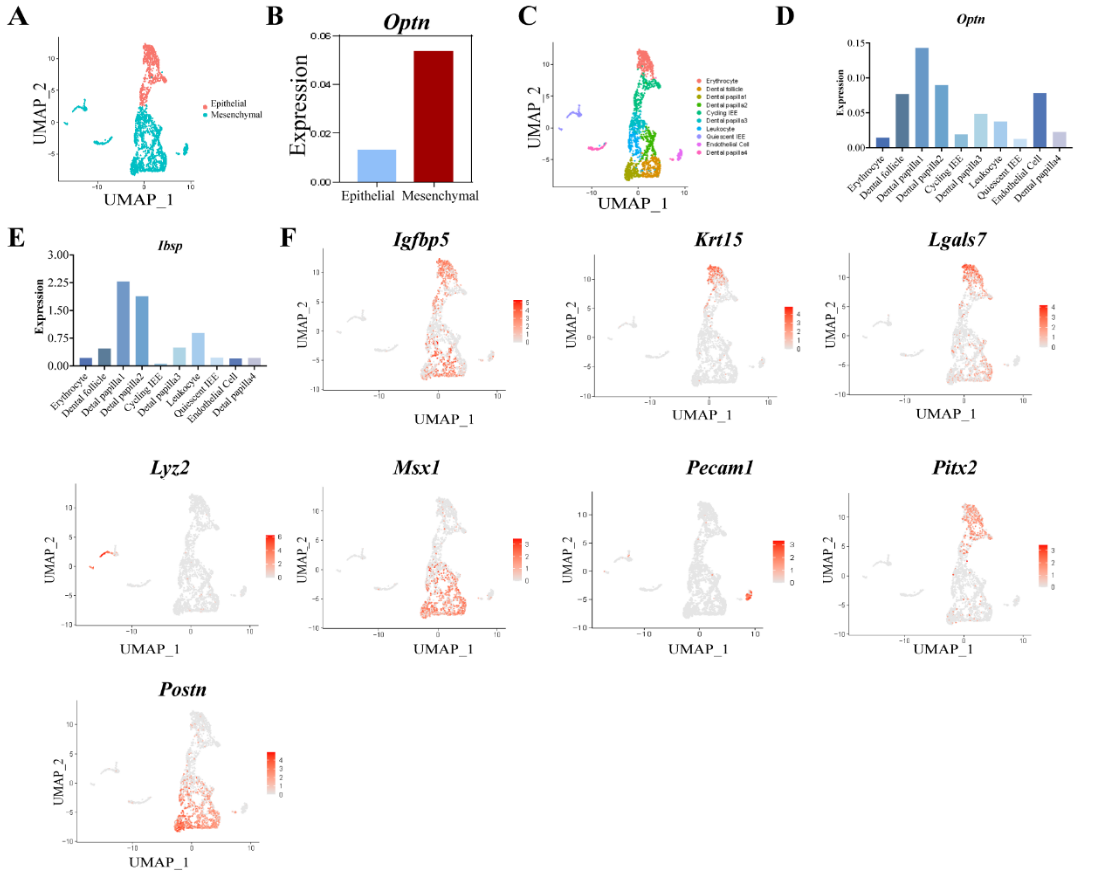

Figure S1. Characterization of gene expression of in mice bell stage tooth germ. (A) Distribution of epithelium and mesenchymal linage cells of mice bell stage tooth germ scRNA-seq analysis on UMAP plot. (B) Average expression of *Optn* between mesenchyme cells and epithelial cells. (C) UMAP of cells divided into different clusters. (D) Average gene expression of *Optn* and *Ibsp* (E) in different clusters. (F) Featureplots indicating marker genes in different clusters, *Igfbp5* for Quiescent IEE, *Krt15* for erythrocyte, *Lgals7* for dental follicle, *Lyz2* for leukocyte, *Msx1* for mesenchymal lineages, *Pecam1* for endothelial cell, *Pitx2* for epithelium linages and cycling IEE, and *Postn* for dental papilla.

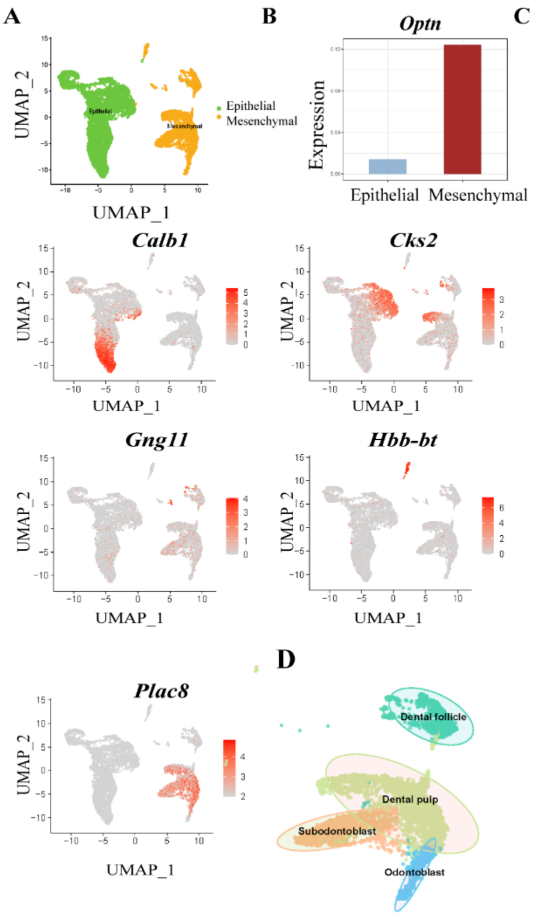

Figure S2. Characterization of gene expression in developing tooth root at P1. (A) UMAP plot of scRNA-seq data sets from mouse P1 molars, showing the distribution from the epithelium and mesenchyme. (B) Average expression of *Optn* between mesenchyme cells and epithelial cells. (C) Featureplots are pseudocolored for the expression of marker genes among different clusters. We identified subodontoblast cluster with *Alpl*, odontoblast with *Bglap*, ameloblast with *Calb1*, cycling iee with *Cks2*, dental follicle with *Col3a1*, quiescent-iee with *Foxp1*, endothelial cell with *Gng1l*, erythrocyte with *Hbb-bt*, preameloblast with *Igfbpl1*, non ameloblast with *Nectin4*, dental pulp with *Plac8*. (D) UMAP of mesenchyme cells, including dental pulp, odontoblasts, subodontoblasts, and dental follicles colored by cell types.

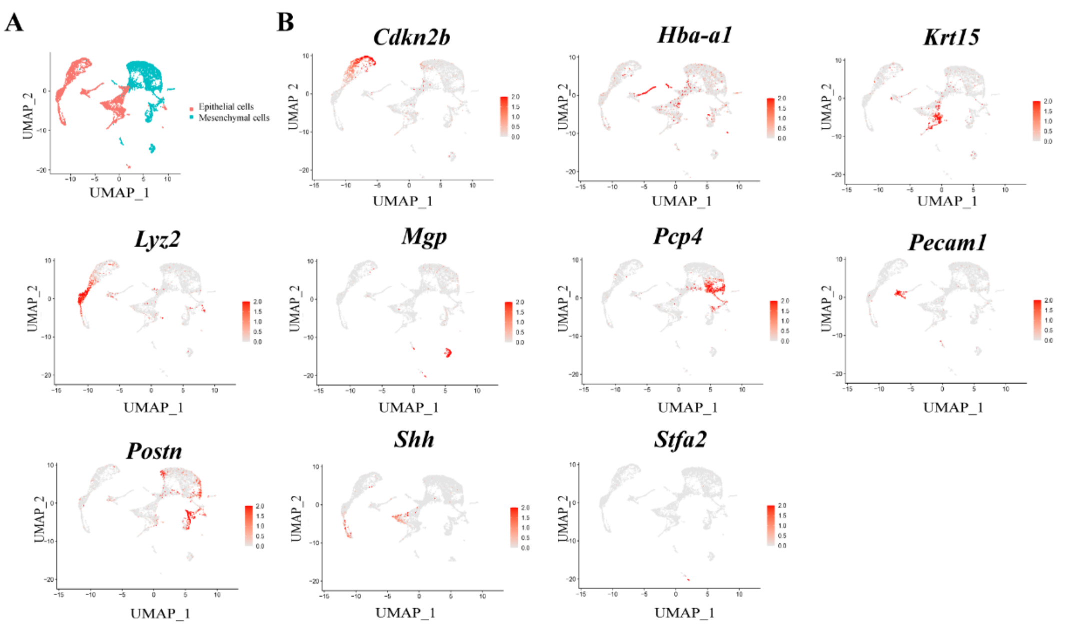

Figure S3. Characterization of gene expression in developing tooth root at P7. (A) UMAP plot of scRNA-seq data sets from mouse P7 molars, showing the distribution from the epithelium and mesenchyme. (B) Featureplots are pseudocolored for the expression of marker genes among different clusters. We identified *Cdkn2b* for IEE, *Hba-a1* for erythroid cells, *Krt15* for SI/SR, *Lyz2* for immune cells, *Mgp* for myoblasts, *Pcp4* for DPC, *Pecam1* for endothelial cells, *Postn* for odontoblasts, *Shh* for EK, and *Stfa2* for Neutrophil.

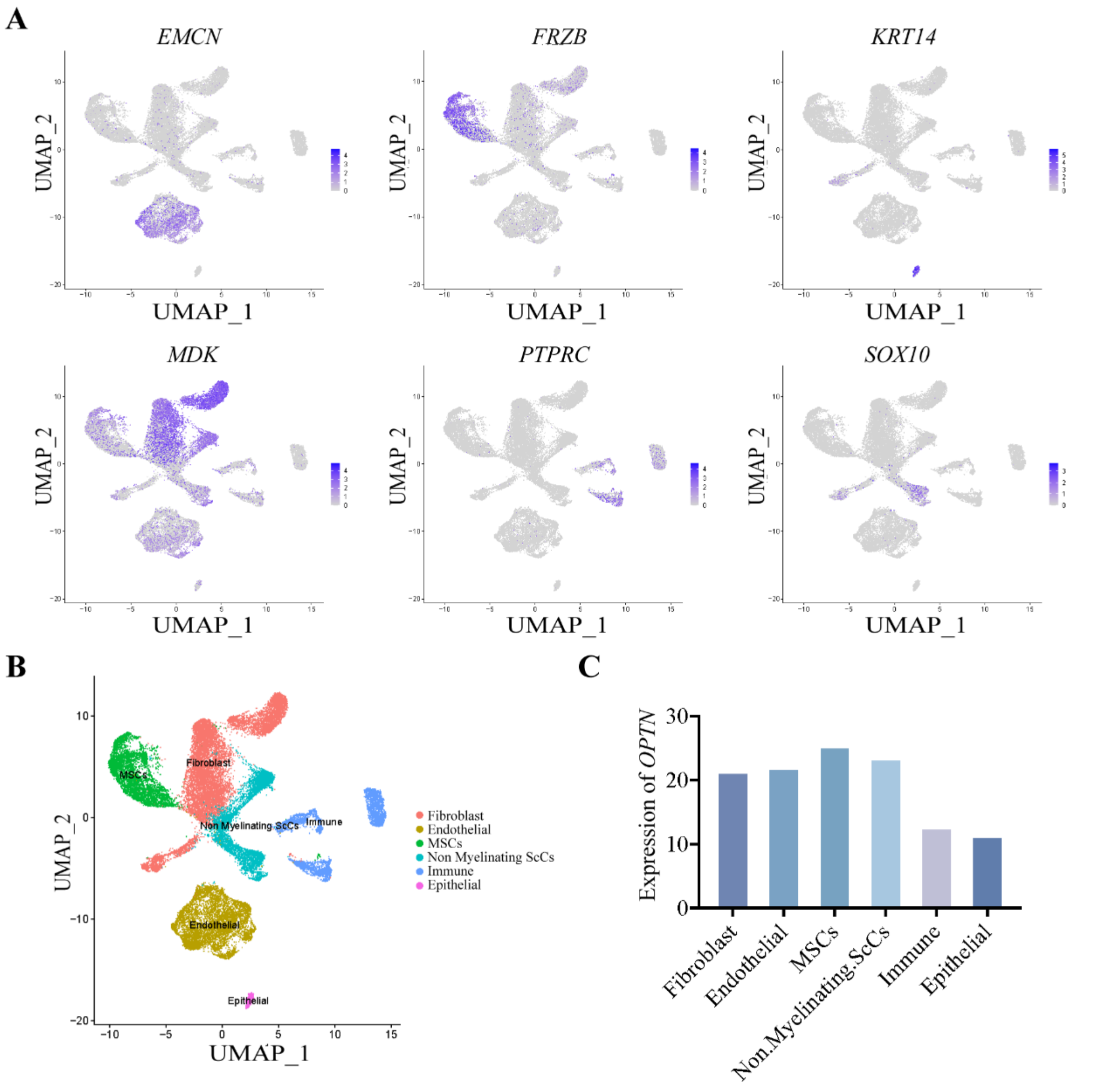

Figure S4. Characterization of gene expression of in human dental pulps. (A) Featureplots indicating marker genes average expression in different clusters, *EMCN* for endothelial cell, *FRZB* for MSCs, *KRT14* for epithlial cells, *MDK* for fibroblast, *PTPRC* for immune cell, *SOX10* for Non Myelinating ScCs. (B) UMAP of cells divided into six different clusters. (C) Average gene expression of *OPTN* in different clusters.

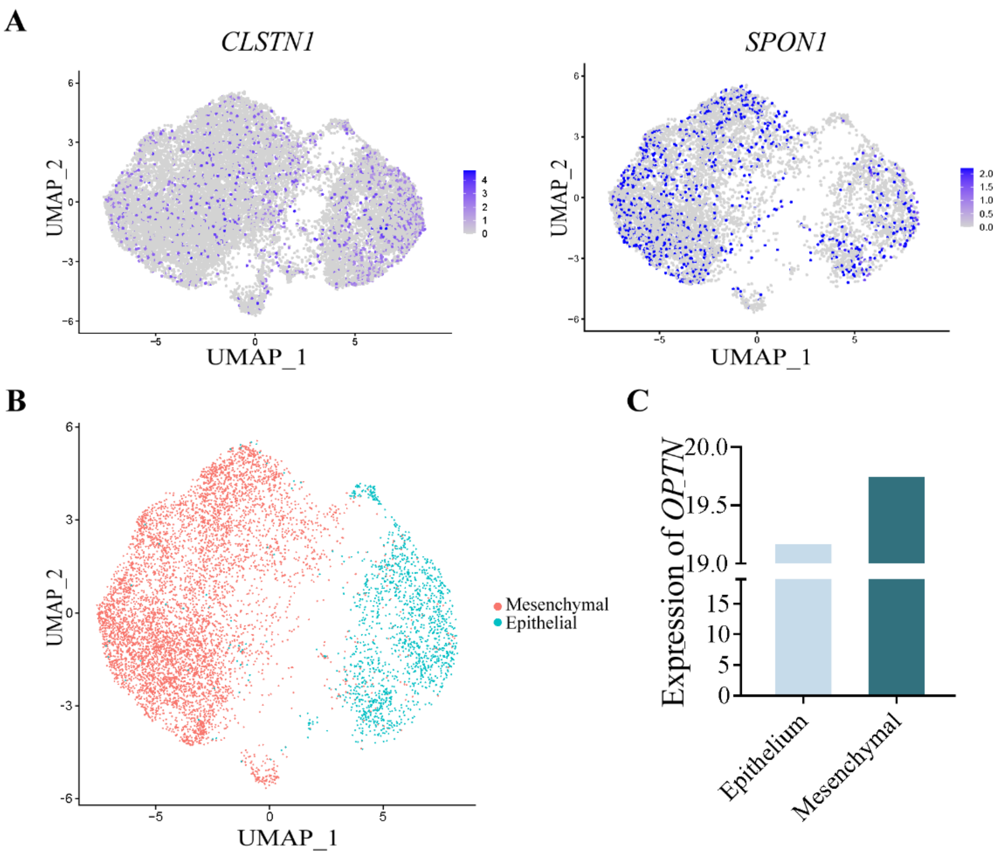

Figure S5. Characterization of gene expression of in human embryonic molars. (A) Featureplots indicating marker genes average expression in different clusters, *CLSTN1* for endothelial cells, *SPON1* for mesenchymal cells. (B) Distribution of epithelial cells and mesenchymal cells of human embryonic molars gern scRNA-seq analysis on UMAP plot. (C)Average expression of *OPTN* between mesenchyme cells and epithelial cells.

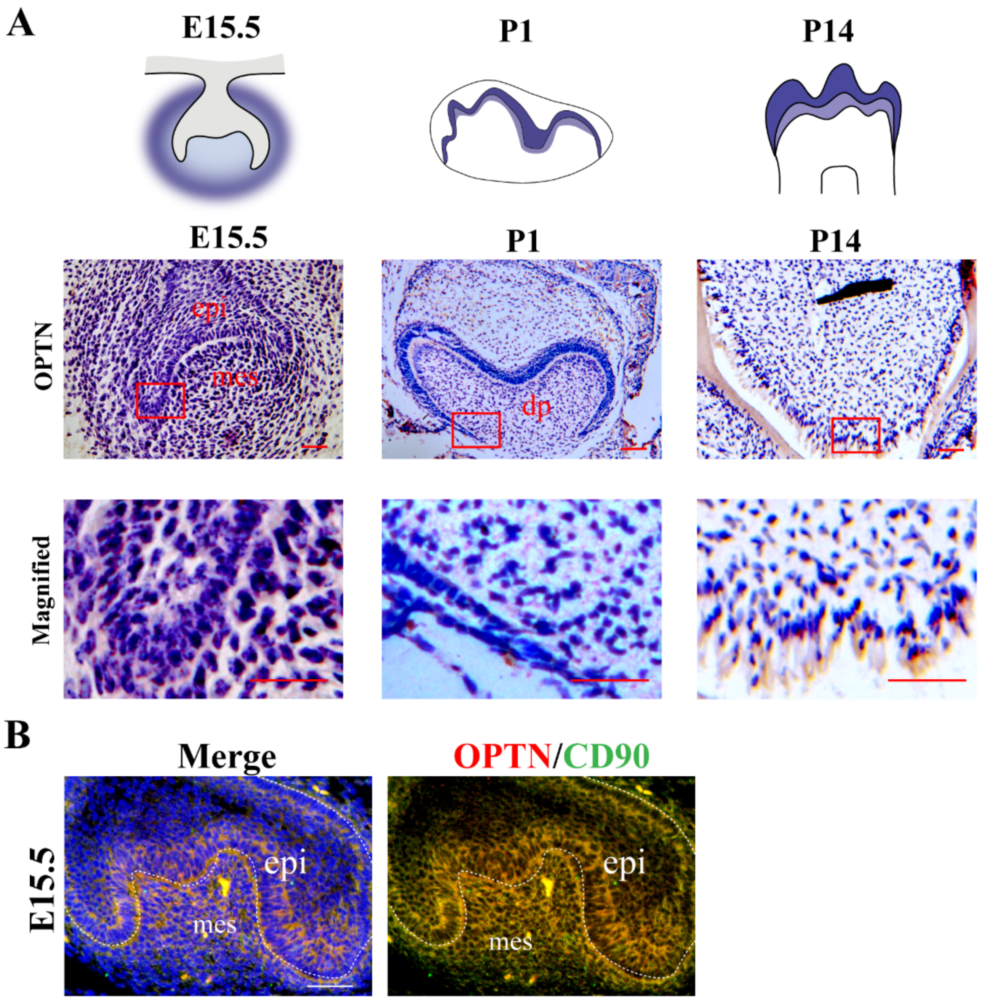

Figure S6. Original expression pattern of OPTN in developing tooth root. (A) Immunohistochemical staining of the OPTN in the developing tooth at E15.5, P1, and P14, respectively. epi: epithelium; mes: mesenchyme; dp: dental papilla. Scale bars: 80μm (up) and 40μm (below). (B) Representative images of immunofluorescent colocalization of OPTN and CD90, with or without DAPI staining in the developing tooth at E15.5. Scale bars: 40μm.

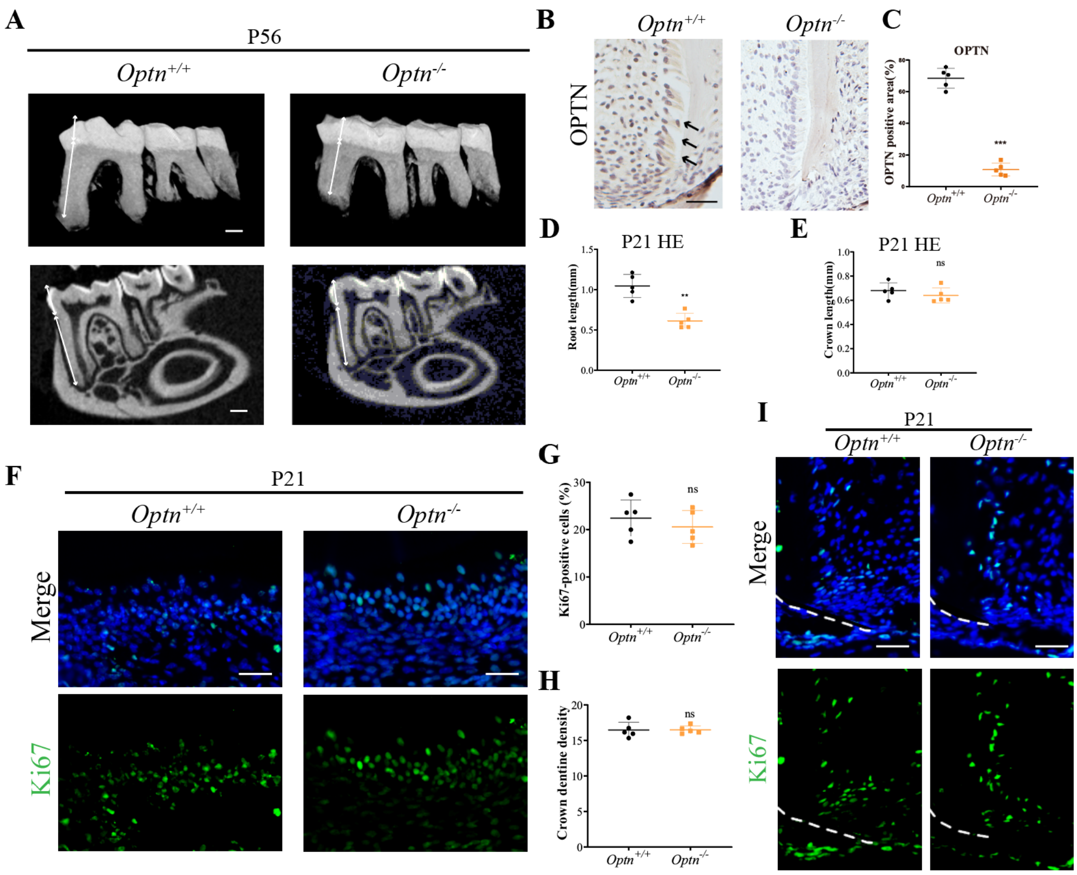

Figure S7. The loss of *Optn* resulted in root development defects. (A) Micro-CT of tooth development in the *Optn*$^{+/+}$ and *Optn*$^{-/-}$ mice at P56. Double-headed arrows indicate tooth crowns and tooth roots. Scale bars: 200µm. (B) Immunohistochemistry staining of apical regions showing the expression pattern of OPTN in the *Optn*$^{+/+}$ and *Optn*$^{-/-}$mice siblings. Arrows indicate the OPTN-positive cells. Scale bars: 50 µm. (C) Quantification of OPTN-positive cells in the apical region is shown in (B). (D, E) Quantitative analysis of the root length and crown length in HE staining of tooth development in the *Optn*$^{+/+}$ and *Optn*$^{-/-}$ mice at P21. (F) Ki67-positive cells in the crown region in the *Optn*$^{+/+}$ and *Optn*$^{-/-}$ mice siblings at P21, Bar=40 µm. (G) Quantification of Ki67-positive cells in (F). (H) Quantification of dentine density in crown region in the *Optn*$^{+/+}$ and *Optn*$^{-/-}$ mice siblings at P21 from CT images. (I) The Ki67-positive

cells in the apical region in the *Optn*$^{+/+}$ and *Optn*$^{-/-}$ mice siblings at P21. Dotted lines indicate the border between the dental epithelium and the dental mesenchyme. The results are presented above as the mean ± SD. SD, Standard Deviation. * $P < 0.05$, ** $P < 0.01$, ***$P < 0.001$, ns, no significance.

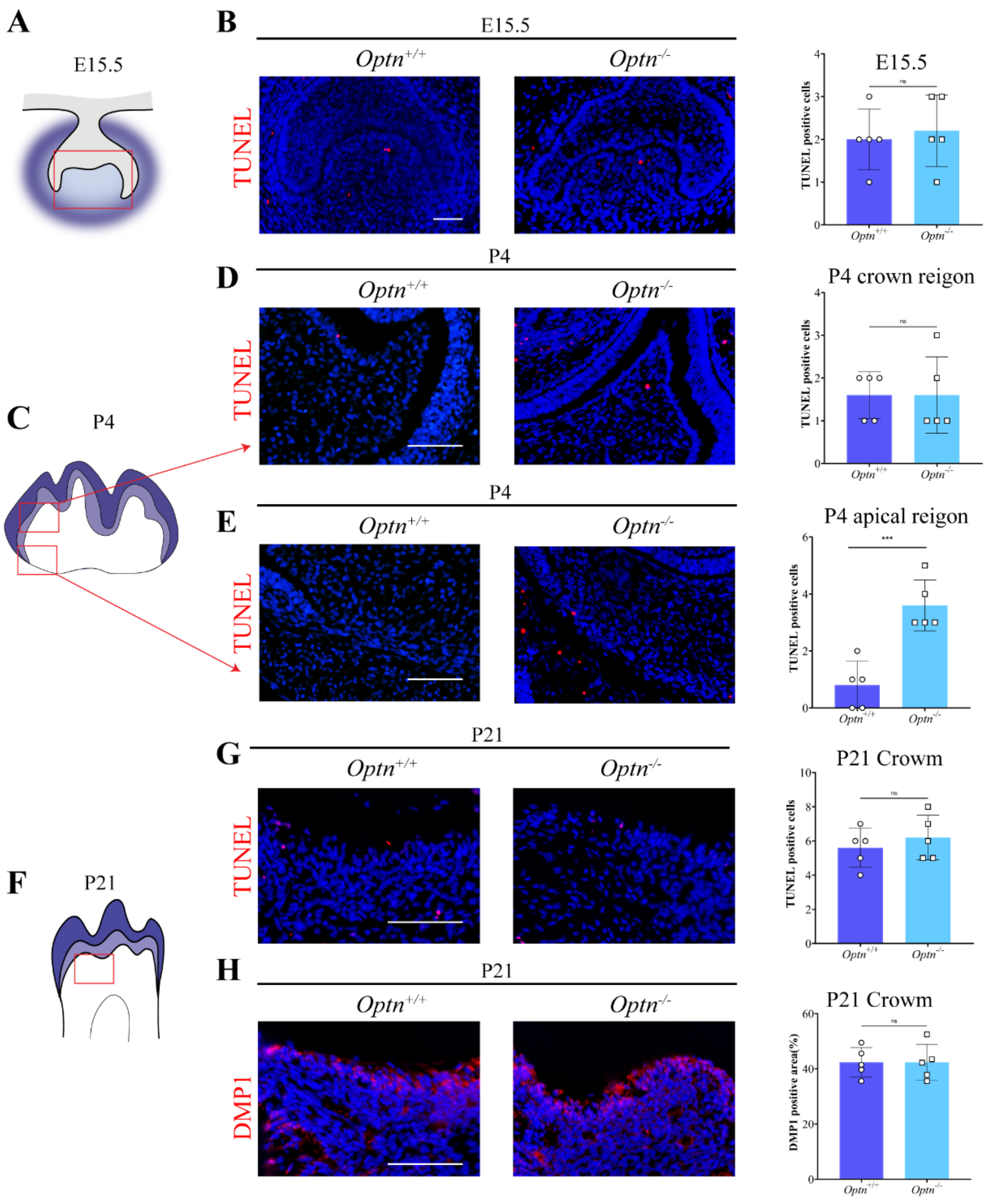

Figure S8. OPTN knockdown barely affects tooth crown development. (A) The schematic diagram of the developing tooth at E15.5. (B) Representative images of TUNEL staining with DAPI staining in the developing tooth were obtained from the *Optn*$^{+/+}$ and *Optn*$^{-/-}$ mice at E15.5 with relative quantification analysis on the right. Scale bars: 80μm. (C) The schematic diagram of the developing tooth at P4 crown region and root region. (D) Representative images of TUNEL staining with DAPI

staining in the developing tooth obtained from the $Optn^{+/+}$ and $Optn^{-/-}$ mice at P4 crown region and root region (E) with relative quantification analysis on the right. Scale bars: 80μm. (F) The schematic diagram of the developing tooth at P21. (G) Representative images of TUNEL staining with DAPI staining in the developing tooth were obtained from the $Optn^{+/+}$ and $Optn^{-/-}$ mice at the P21 crown region with relative quantification analysis on the right. Scale bars: 30μm. (H) Representative images of immunofluorescent staining of DMP1 in the developing tooth obtained from the $Optn^{+/+}$ and $Optn^{-/-}$ mice with relative quantification analysis on the right. Scale bars: 30μm. The results are presented above as the mean ± SD. SD, Standard Deviation. * P < 0.05, ** P < 0.01, ***P < 0.001, ns, no significance.

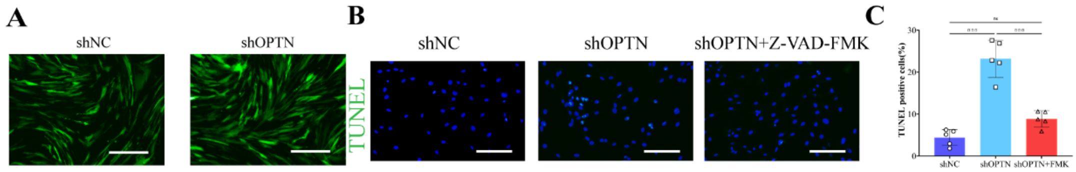

Figure S9. Z-VAD-FMK rescued the cell apoptosis induced by OPTN knockdown. (A) The fluorescence picture of SCAPs infected with shNC or shOPTN lentivirus. Scale bars: 30μm. (B) The appliance of FMK rescued the cell apoptosis in the shOPTN SCAPs. Scale bars: 30μm.(C) The quantification analysis of (B). The results are presented above as the mean ± SD. SD, Standard Deviation. * $P < 0.05$, ** $P < 0.01$, ***$P < 0.001$, ns, no significance.

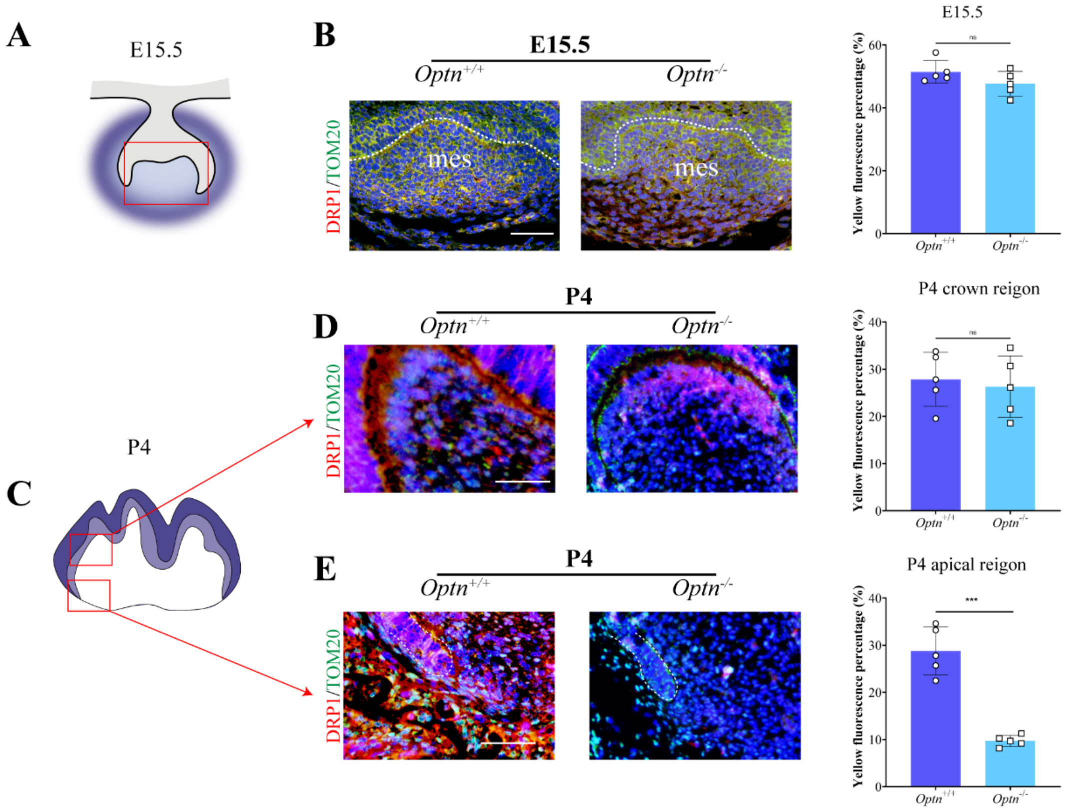

Figure S10. Mitochondria fission was less observed in tooth root region during tooth development. (A) The schematic diagram of the developing tooth at E15.5. (B) Representative images of immunofluorescent colocalization of DRP1 and TOM20, with DAPI staining in the developing tooth at E15.5, with relative quantification analysis on the right. (C) The schematic diagram of the crown region and the apical region of developing tooth at P4. (D) Representative images of immunofluorescent colocalization of DRP1 and TOM20, with DAPI staining in the crown reigon and the apical region (E) at P4 from obtained from *Optn*$^{+/+}$ and *Optn*$^{-/-}$ mice with relative quantification analysis on the right. Scale bars: 80μm. The results are presented above as the mean ± SD. SD, Standard Deviation. * $P < 0.05$, ** $P < 0.01$, ***$P < 0.001$, ns, no significance.

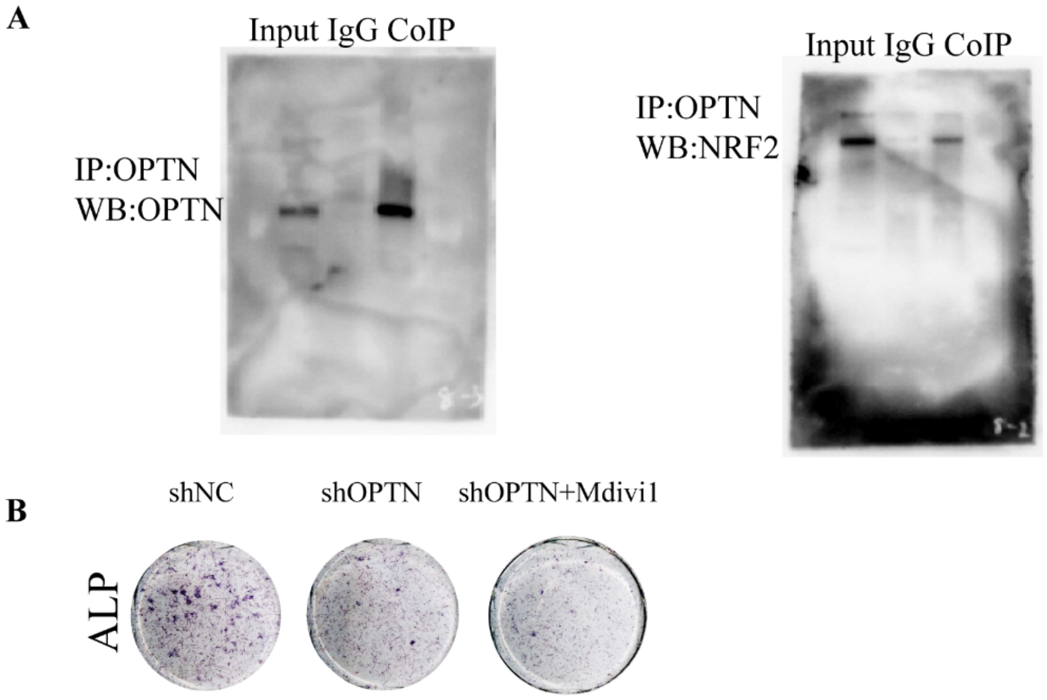

Figure S11. Decreasing phosphorylation of DRP1 worsens the decreased ALP activity in the shOPTN SCAPs. (A) The full loading band of CO-IP of OPTN with NRF2. (B) The appliance of Mdivi-1 decreased the ALP activity in the shOPTN SCAPs.

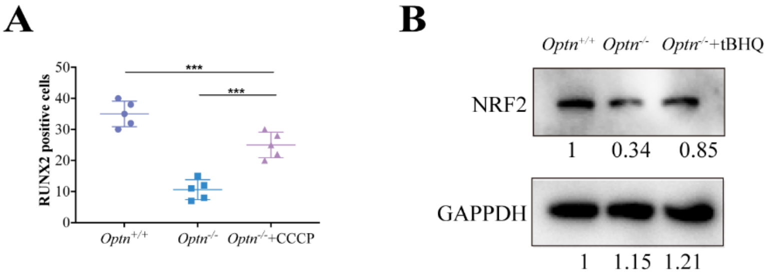

Figure S12. Subgingival injections rescued tooth root development. (A) The quantification of RUNX2-positive cells within the roots of the mandibular first molars, obtained from the *Optn*$^{+/+}$, *Optn*$^{-/-}$, and *Optn*$^{-/-}$ +CCCP group following subgingival injections. (B) The WB shows the succeseful appliance of tBHQ. The results are presented above as the mean ± SD. SD, Standard Deviation. * $P < 0.05$, ** $P < 0.01$, ***$P < 0.001$, ns, no significance.

Table S2: Lists of top 200 genes up/down-regulated in shOPTN SCAPs compared with shNC SCAPs (log2-fold changel> 0.584 and p-value < 0.05).

gene_id	gene_name	EntrezID	B_1	B_2	B_3	A_1	A_2	A_3	pval	qval	fc	log2(fc)	regulation	significant
ENSG00000181195	PENK	5179	53.8020643	52.7164983	53.0173468	12.7855368	15.9729981	13.9237307	7.422E-110	2.405E-105	3.73775636	1.90217253	up	yes
ENSG00000120738	EGR1	1958	58.456051	86.069626	72.601799	23.51516	23.063789	22.768581	5.154E-53	8.3489E-49	3.13100519	1.6466259	up	yes
ENSG00000171570	RAB4B-EGLN2	100529264	0.09208047	0.06273616	0.07327422	3.5699286	1.57589272	3.61266729	4.5009E-52	4.8607E-48	0.02604226	-5.2630015	down	yes
ENSG00000170345	FOS	2353	4.66291121	4.80079631	4.41031053	1.10586957	1.09600366	0.97227101	1.5581E-47	1.262E-43	4.37094757	2.12794607	up	yes
ENSG00000229124	VIM-AS1	100507347	7.00523755	9.78469394	8.02095573	30.1017156	43.8061862	29.6765238	3.4748E-46	2.2515E-42	0.23952334	-2.06176187	down	yes
ENSG00000073756	PTGS2	5743	3.40960732	2.75274689	3.34627645	0.72855415	0.47701661	0.60712694	5.7586E-45	3.1095E-41	5.24556889	2.39109924	up	yes
ENSG00000259001	ENSG00000259001		169.369202	110.844093	149.285767	44.683563	34.346611	40.911938	1.0813E-44	5.0045E-41	3.58088627	1.8403167	up	yes
ENSG00000120708	TGFBI	7045	29.9793735	31.339663	31.2980602	17.0920977	16.038095	16.4314139	1.4393E-44	5.8288E-41	1.86872668	0.90205558	up	yes
ENSG00000110900	TSPAN11	441631	19.3733038	15.9749822	17.1226084	8.44082579	8.31674545	8.31650158	1.8985E-38	6.8343E-35	2.09263548	1.06532103	up	yes
ENSG00000181019	NQO1	1728	79.0884155	80.8481888	77.5425302	43.8886172	47.6884264	44.3008789	1.4472E-34	4.6886E-31	1.74773893	0.8054897	up	yes
ENSG00000123240	OPTN	10133	10.746359	10.9206957	11.235489	19.7180447	21.0886872	21.2520774	7.6902E-34	2.265E-30	0.53018329	-0.91543689	down	yes
ENSG00000095303	PTGS1	5742	55.0143534	50.225198	52.659563	32.550752	30.0176692	31.4218262	1.0936E-29	2.9525E-26	1.67995211	0.74842011	up	yes
ENSG00000116741	RGS2	5997,6012	50.7348596	35.6977018	42.457481	19.4677306	14.9339686	16.5024188	5.4276E-29	1.3526E-25	2.53201603	1.34028654	up	yes
ENSG00000072274	TFRC	7037	10.7728388	9.76485855	10.0170256	5.98360004	6.05454185	6.18792398	1.3616E-27	3.151E-24	1.67642997	0.74539222	up	yes
ENSG00000146250	PRSS35	167681	38.85944	26.371082	30.885368	14.047457	11.220821	12.699424	1.6979E-27	3.6673E-24	2.53151718	1.34000228	up	yes
ENSG00000011105	TSPAN9	10867	49.9515604	50.1631737	48.9933847	32.3574262	28.1667494	29.3802966	2.1085E-27	4.2695E-24	1.65851726	0.72989403	up	yes
ENSG00000186469	GNG2	54331	16.7893247	14.9722278	15.4959028	8.71708308	8.7589753	9.14803737	2.8123E-27	5.3596E-24	1.77498818	0.82780942	up	yes
ENSG00000123358	NR4A1	3164	4.13254632	3.66194174	4.32104	1.57731852	1.62422551	1.85670649	1.9153E-26	3.4473E-23	2.39520126	1.26014689	up	yes
ENSG00000137975	CLCA2	9635	3.51308691	2.08276103	2.78107599	0.68584746	0.33311302	0.44725482	3.238E-25	5.5213E-22	5.71329732	2.51432361	up	yes
ENSG00000141526	SLC16A3	9123	14.0452792	12.5872996	13.4927474	8.09775661	8.06500086	7.72421481	3.698E-25	5.9904E-22	1.67979959	0.74828912	up	yes
ENSG00000110092	CCND1	595	65.1592798	64.9480804	64.2483786	97.197969	97.1990962	97.9975264	3.0497E-24	4.7049E-21	0.6647036	-0.58921692	down	yes

ENSG00000168398	BDKRB2	624	17.0553543	10.8435847	13.1325988	5.4613932	5.8727335	5.89969837	6.2244E-24	9.1662E-21	2.38087237	1.25149028	up	yes
ENSG00000163531	NFASC	23114	1.28150794	1.33556491	1.34937254	2.38360135	3.04351145	2.67691117	1.2975E-23	1.8277E-20	0.48944147	-1.03079175	down	yes
ENSG00000129946	SHC2	25759	0.48021705	0.49313737	0.475887	1.97023816	1.7378078	1.7133657	4.6521E-23	6.28E-20	0.26731809	-1.90337061	down	yes
ENSG00000125730	C3	718	5.96304009	3.41071523	4.6612337	1.41599022	1.86708661	1.62182571	1.7164E-22	2.2243E-19	2.86142057	1.51673156	up	yes
ENSG00000115594	IL1R1	3554	232.08593	191.004145	207.116068	133.589534	120.735197	125.332788	3.3396E-22	4.1614E-19	1.65993326	0.73112524	up	yes
ENSG00000172379	ARNT2	9915	3.80121636	4.67743464	3.73317245	8.05978031	9.03385192	7.51024744	2.7129E-21	3.2553E-18	0.49633731	-1.01060719	down	yes
ENSG00000096696	DSP	1832,1834	1.70833364	1.86648375	1.56865901	3.08465444	3.44633371	3.20654163	2.4589E-20	2.8024E-17	0.52821162	-0.92081205	down	yes
ENSG00000169429	CXCL8	3576	2.4936403	2.61511957	2.71673782	0.61594606	0.68280487	0.36507074	2.5085E-20	2.8024E-17	4.70332721	2.2336817	up	yes
ENSG00000126803	HSPA2	3306	12.4371049	10.5131789	11.0534891	6.28539562	6.68292672	6.32218612	2.8797E-20	3.1099E-17	1.7627204	0.81780366	up	yes
ENSG00000000971	CFH	3075	39.4890485	31.6769673	33.2145898	20.2401556	21.5676324	21.1762288	1.8616E-19	1.9455E-16	1.65725546	0.728796	up	yes
ENSG00000175899	A2M	2,3494	1.55225237	1.04714804	0.95179046	0.20084692	0.25183402	0.30741116	3.9036E-19	3.9522E-16	4.67205334	2.22405674	up	yes
ENSG00000089597	GANAB	23193	86.511397	85.5713285	81.4429671	59.7698977	59.0196755	55.1138998	4.7727E-19	4.6856E-16	1.45785296	0.54384521	up	no
ENSG00000118515	SGK1	6446	17.1543312	17.9062951	17.3885444	10.7645	6.97504865	8.50098987	5.755E-19	5.4838E-16	1.99878408	0.99912263	up	yes
ENSG00000171223	JUNB	3726,10535	39.052315	40.912731	37.934898	23.164331	14.783875	16.185043	8.9432E-19	8.2784E-16	2.17795802	1.12297615	up	yes
ENSG00000064393	HIPK2	28996	25.9701034	24.2831263	24.1555199	14.6857572	17.2078569	15.7574217	1.7425E-18	1.5682E-15	1.56153478	0.6429647	up	yes
ENSG00000119699	TGFB3	7043	16.2395057	15.3713106	15.4661274	10.2253455	7.77284449	8.27564513	5.4099E-18	4.7371E-15	1.79178044	0.84139386	up	yes
ENSG00000070214	SLC44A1	23446	34.9000117	31.1585268	32.6502169	23.0659898	20.8672748	21.7852058	5.6047E-18	4.7785E-15	1.50199411	0.58687916	up	yes
ENSG00000142949	PTPRF	5792	2.35307424	3.01537229	2.83373587	5.05090818	5.57867012	4.90594049	6.323E-18	5.2526E-15	0.52796321	-0.92149069	down	yes
ENSG00000135842	NIBAN1	116496	7.90975649	10.771922	8.77316129	16.3736915	16.9311208	16.3917849	9.2424E-18	7.4859E-15	0.55244909	-0.85608658	down	yes
ENSG00000140945	CDH13	1012	8.2552545	8.58789137	8.22933247	13.0602158	12.2112745	12.2100733	1.9053E-17	1.5055E-14	0.66892829	-0.58007653	down	no
ENSG00000019991	HGF	3082,3569,6654	14.6496419	10.8775735	12.1050428	6.93208923	6.17456448	7.33031283	2.0662E-17	1.5938E-14	1.8413818	0.88078879	up	yes
ENSG00000134853	PDGFRA	5156	184.140004	163.943783	164.038895	118.674219	110.929701	118.202557	3.0391E-17	2.2898E-14	1.47243573	0.55820466	up	no
ENSG00000163453	IGFBP7	3490	164.032831	153.531992	169.541804	113.153925	95.4427521	88.9047975	3.8197E-17	2.8125E-14	1.63732509	0.71134079	up	yes
ENSG00000169744	LDB2	9079	9.05226283	8.40140762	8.92350679	5.66830584	4.9601256	4.9122509	5.4412E-17	3.9174E-14	1.69729853	0.76324033	up	yes
ENSG00000168385	SEPTIN2	4735	69.768611	68.4058985	66.2453869	49.9315774	48.6681669	48.3495657	6.3775E-17	4.4917E-14	1.39109123	0.47621704	up	no
ENSG00000152402	GUCY1A2	2977	0.49313063	0.55358072	0.49572458	1.11320293	1.00708611	1.08429468	7.6632E-17	5.2824E-14	0.48132178	-1.0549	down	yes

											264		
ENSG00000185567	AHNAK2	113146	3.45159472	4.06853983	3.52725095	5.95093707	7.93837131	6.41167959	8.8383E-17	5.9655E-14	0.5441797	-0.87784496	down	yes
ENSG00000131389	SLC6A6	6533	25.2478199	23.6390211	23.4886813	16.3271438	11.6156526	12.8822119	1.0118E-16	6.6896E-14	1.77282321	0.82604868	up	yes
ENSG00000106948	AKNA	80709	2.43467068	2.41958981	2.4833647	4.1548579	3.89380953	4.02728644	1.5613E-16	1.0117E-13	0.60762282	-0.71875203	down	yes
ENSG00000130203	APOE	348	22.3827087	17.4048313	18.9476468	11.0161557	10.9322928	10.0340329	2.2175E-16	1.4086E-13	1.83647998	0.87694317	up	yes
ENSG00000163739	CXCL1	2919	5.67293524	3.54865343	4.75892006	1.40029797	1.08700197	1.04161319	3.1393E-16	1.9559E-13	3.96170385	1.98612104	up	yes
ENSG00000164188	RANBP3L	202151	3.30259822	3.17168858	3.09845878	1.61937328	1.23561191	1.03398955	6.1075E-16	3.7334E-13	2.46150881	1.2995429	up	yes
ENSG00000122642	FKBP9	11328	32.8907999	31.5865078	32.2447075	45.369656	53.8602652	48.8290302	1.2283E-15	7.3691E-13	0.65326692	-0.61425551	down	yes
ENSG00000160963	COL26A1	136227	1.33291123	1.48091744	1.65653295	0.43041197	0.29517834	0.33230389	1.2625E-15	7.4369E-13	4.2257171	2.07919619	up	yes
ENSG00000008256	CYTH3	9265	11.9478432	15.4231456	13.8901823	22.1544762	26.5052562	23.2294424	1.4298E-15	8.2718E-13	0.57395527	-0.80098978	down	yes
ENSG00000139324	TMTC3	160418	9.02268157	8.92805088	8.93863398	5.16825295	4.77666055	5.96887125	1.4625E-15	8.3127E-13	1.68969022	0.75675877	up	yes
ENSG00000120659	TNFSF11	8600	1.79324258	0.92277852	1.38907206	0.23551122	0.16670035	0.23241189	1.5427E-15	8.6173E-13	6.4685493	2.6934422	up	yes
ENSG00000113369	ARRDC3	57561	8.72373836	6.3604141	7.19324924	3.85452273	3.29854113	4.02910518	2.8269E-15	1.5523E-12	1.99222545	0.99438092	up	yes
ENSG00000101187	SLCO4A1	28231	5.14856016	6.05688052	5.24794956	2.42377938	1.10635816	2.00927596	4.4611E-15	2.4088E-12	2.97024048	1.57057974	up	yes
ENSG00000108946	PRKAR1A	5573	69.6612235	52.7467797	59.4221229	39.9229726	33.1647838	36.9482827	6.3782E-15	3.3689E-12	1.65245975	0.72461513	up	yes
ENSG00000164171	ITGA2	3673	15.9985787	18.621587	16.1621186	10.4138808	6.49451	9.01907048	6.4471E-15	3.3689E-12	1.95862926	0.96984434	up	yes
ENSG00000128849	CGNL1	84952	3.59751417	3.10358988	3.18848462	1.98730011	1.7448024	1.57449584	7.4946E-15	3.8541E-12	1.86363995	0.89812316	up	yes
ENSG00000233695	GAS6-AS1	650669	0.85442318	1.04605513	0.73627395	2.08630021	1.89748807	2.10907148	9.7425E-15	4.7219E-12	0.43276103	-1.2083575	down	yes
ENSG00000176438	SYNE3	161176	2.73438412	4.15253609	3.90291117	6.40787287	7.73465412	7.01283638	9.7651E-15	4.7219E-12	0.51002818	-0.97135115	down	yes
ENSG00000101825	MXRA5	25878	62.941483	55.625713	57.585663	40.234516	40.006123	42.41275	9.5502E-15	4.7219E-12	1.4361842	0.52224079	up	no
ENSG00000185885	IFITM1	8519	118.545335	78.6580122	98.6609187	55.2771688	55.9953122	52.3267197	9.4312E-15	4.7219E-12	1.80847012	0.85476976	up	yes
ENSG00000197930	ERO1A	30001	8.60535222	8.73503523	8.37993018	6.00573097	5.11399761	5.26639416	1.274E-14	6.0697E-12	1.56964024	0.65043393	up	yes
ENSG00000102996	MMP15	4324	1.71678687	2.43423342	2.55598398	5.07053575	5.3920357	4.68055126	1.3022E-14	6.1145E-12	0.44290761	-1.17492231	down	yes
ENSG00000176907	TCIM	56892	5.880254	4.126458	4.318706	1.638946	1.40543	1.843617	1.8734E-14	8.6706E-12	2.93073619	1.55126311	up	yes
ENSG00000156011	PSD3	23362	11.0270807	9.68964189	9.58794335	6.6881794	6.8952188	6.8965416	2.4288E-14	1.1083E-11	1.47972436	0.56532846	up	no
ENSG00000151176	PLBD2	196463	25.115804	23.539651	24.8165665	34.0003166	36.6463412	35.1058381	2.8567E-14	1.2854E-11	0.69475449	-0.52542484	down	no

ENSG00000143369	ECM1	1893	36.5231441	37.9627312	37.0477991	50.9944134	54.2761479	53.6959442	3.7078E-14	1.6233E-11	0.70161745	-0.51124346	down	no
ENSG00000184205	TSPYL2	64061	11.753638	10.4390151	10.4895847	7.0255667	7.14255307	6.77358658	3.6754E-14	1.6233E-11	1.56062918	0.64212778	up	yes
ENSG00000150681	RGS18	64407	2.68380608	0.5480332	1.2420709	0.12341355	0.13910681	0.15744823	4.353E-14	1.8804E-11	10.6529637	3.41318295	up	yes
ENSG00000125637	PSD4	23550	2.55982484	2.18275941	2.33675527	1.38370568	1.4468846	1.308235	4.8795E-14	2.0801E-11	1.71047074	0.77439342	up	yes
ENSG00000160932	LY6E	4061	96.9440322	87.2421913	94.4444841	61.5753531	66.4989929	65.4760707	7.6425E-14	3.2156E-11	1.43957689	0.52564485	up	no
ENSG00000152558	TMEM123	114908,10360	25.2324468	23.1031472	23.1913117	33.3885903	34.1391494	34.2815705	2.1526E-13	8.9411E-11	0.70255761	-0.50931156	down	no
ENSG00000026025	VIM	7431	1601.37173	1779.84851	1703.66814	2279.83748	2428.50903	2418.78585	2.4238E-13	9.9401E-11	0.71345502	-0.48710561	down	no
ENSG00000163661	PTX3	5806,5309	51.978645	72.393196	62.874233	101.782486	117.836411	100.911873	2.6181E-13	1.0603E-10	0.58417504	-0.77552739	down	yes
ENSG00000170801	HTRA3	94031,726	22.7029046	17.0651984	20.5216239	12.3973517	10.2242735	12.0010475	3.8103E-13	1.524E-10	1.74133659	0.8001951	up	yes
ENSG00000087303	NID2	22795	13.1790219	11.6667733	11.9369036	6.58014318	8.44314363	7.47536688	6.159E-13	2.4334E-10	1.63488444	0.70918866	up	yes
ENSG00000114948	ADAM23	8745	1.03815295	0.90908513	0.87110444	2.30184113	1.78155624	1.99716506	6.6302E-13	2.588E-10	0.4635003	-1.10935782	down	yes
ENSG00000170214	ADRA1B	147	3.98613922	5.65001597	5.56691623	9.31059516	11.3077283	9.8108101	7.3837E-13	2.8435E-10	0.49962222	-1.00109044	down	yes
ENSG00000170412	GPRC5C	55890	0.85652049	0.57548797	0.82382589	0.23563061	0.19905775	0.24247175	7.4603E-13	2.8435E-10	3.33131611	1.73609226	up	yes
ENSG00000099194	SCD	6319	77.87101	71.245323	73.366707	101.445175	99.70356	103.976334	7.5866E-13	2.858E-10	0.72915359	-0.45570535	down	no
ENSG00000104321	TRPA1	8989	20.1433907	14.6892501	17.2344127	10.7999671	9.21276309	10.7890363	9.1712E-13	3.4153E-10	1.69039179	0.75735767	up	yes
ENSG00000070756	PABPC1	26986	63.5937304	65.2289876	62.7457683	84.5792122	88.8708764	96.2946107	1.053E-12	3.8767E-10	0.71018443	-0.49373436	down	no
ENSG00000259207	ITGB3	3690	4.93054812	6.13615253	4.50952017	2.71366317	3.13364926	2.44959106	1.1895E-12	4.3302E-10	1.8773535	0.90870033	up	yes
ENSG00000085662	AKR1B1	231	36.6905993	29.4521934	30.6828791	21.951149	17.2528011	19.9705287	1.3118E-12	4.722E-10	1.63627418	0.71041451	up	yes
ENSG00000100234	TIMP3	7078	137.533157	134.612244	133.449982	99.554337	85.426277	99.944038	1.3945E-12	4.9649E-10	1.42351804	0.50946078	up	no
ENSG00000152952	PLOD2	5352	28.5255126	27.0266604	27.1959719	20.5094863	16.3423339	18.6813835	1.7811E-12	6.2723E-10	1.49006611	0.57537634	up	no
ENSG00000065534	MYLK	4638	5.32450672	6.09375651	5.46691147	7.7105393	9.4111239	8.61348656	1.8807E-12	6.5517E-10	0.65611333	-0.60798307	down	yes
ENSG00000136960	ENPP2	5168	16.1570824	11.2101856	12.3183607	7.94371497	7.90875295	6.92853003	2.0055E-12	6.9121E-10	1.74204961	0.80078571	up	yes
ENSG00000165806	CASP7	840	9.69473109	8.85903485	9.12097394	5.79008302	6.09128788	5.3844934	2.0797E-12	7.0925E-10	1.60285865	0.6806472	up	yes
ENSG00000221890	NPTXR	23467	1.684873	2.197985	1.838188	3.534238	3.539678	3.658247	2.1167E-12	7.1435E-10	0.53307483	-0.90759002	down	yes
ENSG0000	TGM2	7052	3.9281	3.2000	3.3923	2.0426	2.0692	2.0844	3.3634	1.1234	1.6978	0.7637	up	yes

0198959			5195	5144	5842	1011	4101	8655	E-12	E-09	6774	2408		
ENSG00000113583	C5orf15	56951	39.4205833	36.1412186	34.0310262	26.1332608	24.1616192	24.5897149	4.3216E-12	1.4287E-09	1.46348963	0.54941252	up	no
ENSG00000070193	FGF10	2255	0.48999032	0.52790308	0.59822908	1.14979767	1.34304012	1.4360788	4.366E-12	1.4288E-09	0.41134049	-1.281595	down	yes
ENSG00000166598	HSP90B1	7184	35.2384012	32.6023525	31.9758007	25.2192166	23.8207999	24.7585044	4.662E-12	1.5104E-09	1.35255495	0.4356872	up	no
ENSG00000134871	COL4A2	1284	14.7963591	16.3478671	15.3748861	11.6674249	9.2015699	10.310037	5.4489E-12	1.7479E-09	1.4919999	0.57724744	up	no
ENSG00000150867	PIP4K2A	5305	7.31626085	8.51669753	7.83588238	12.3767849	11.8938523	11.6089575	5.7516E-12	1.8269E-09	0.65967414	-0.60017454	down	yes
ENSG00000128591	FLNC	2318	14.8568399	19.7465971	17.067256	25.2491375	29.6001323	27.7956053	5.9314E-12	1.8487E-09	0.62521352	-0.67757912	down	yes
ENSG00000138829	FBN2	2201	169.565745	220.193031	187.295154	283.768929	294.512493	287.953306	5.9345E-12	1.8487E-09	0.66616347	-0.58605186	down	yes
ENSG00000125257	ABCC4	10257	9.84734408	9.79506651	9.27700369	7.15752907	6.49146705	6.65405331	6.1736E-12	1.8997E-09	1.42438772	0.5103419	up	no
ENSG00000138944	SHISAL1	85352	4.22759	2.845098	3.362513	1.987758	1.530182	1.517979	6.2155E-12	1.8997E-09	2.07215426	1.05113141	up	yes
ENSG00000196526	AFAP1	60312	10.4402969	10.9932793	10.4503116	7.8181784	7.57535316	7.6844093	7.1947E-12	2.1784E-09	1.3815742	0.46631305	up	no
ENSG00000169851	PCDH7	5099	0.81576593	0.95312597	0.98596995	0.42457786	0.38500665	0.48253292	8.1302E-12	2.4389E-09	2.13205223	1.09224278	up	yes
ENSG00000121039	RDH10	157506	8.00301692	5.05280893	6.81310069	3.2732504	2.6313187	3.65770727	8.3996E-12	2.4966E-09	2.07784483	1.05508792	up	yes
ENSG00000179218	CALR	811	241.715029	215.337716	222.580807	168.137062	167.647746	168.758586	9.9645E-12	2.9348E-09	1.34702695	0.42977872	up	no
ENSG00000102931	ARL2BP	23568	26.4842936	25.2748423	25.9532581	16.3634799	18.3557379	17.3595091	1.134E-11	3.2803E-09	1.49220994	0.57745052	up	no
ENSG00000140563	MCTP2	55784	3.29659084	2.72361263	2.60387887	1.82548446	1.27113626	1.57004385	1.1272E-11	3.2803E-09	1.84801847	0.88597918	up	yes
ENSG00000157613	CREB3L1	90993	19.8222977	26.3416207	23.6575602	33.4310543	40.6872057	42.946687	1.8051E-11	5.1753E-09	0.59643369	-0.74556634	down	yes
ENSG00000125968	ID1	3397	21.9254612	13.4817838	16.3160417	7.9229517	8.65244279	8.24376426	2.2895E-11	6.5067E-09	2.08400644	1.05935974	up	yes
ENSG00000072041	SLC6A15	55117	0.60189087	0.43139011	0.59878518	0.26981469	0.13973645	0.15387957	2.3509E-11	6.623E-09	2.89665812	1.53438942	up	yes
ENSG00000069869	NEDD4	4734	14.0828014	14.3682095	13.4144844	9.99013238	10.3296006	10.33716	2.6094E-11	7.2878E-09	1.36561443	0.44955021	up	no
ENSG00000272636	DOC2B	8447	1.08926549	0.873738	0.97011845	0.41486145	0.30458877	0.38524648	2.7469E-11	7.6063E-09	2.65513779	1.40878673	up	yes
ENSG00000174136	RGMB	285704	11.8223691	13.4645903	12.3625013	17.5906947	17.7984354	18.1937861	2.9016E-11	7.9666E-09	0.70263926	-0.5091439	down	no
ENSG00000058091	CDK14	5218	5.92916962	5.08100455	5.08857776	2.85451632	3.44106357	3.4169492	3.4003E-11	9.2574E-09	1.65752419	0.72902993	up	yes
ENSG00000159322	ADPGK	83440	9.81193091	9.01000798	8.49053341	6.15710521	6.12112003	6.2593361	3.542E-11	9.5628E-09	1.47335843	0.55910844	up	no
ENSG00000029364	SLC39A9	55334	10.6920612	10.134781	9.81518702	7.11335378	6.35338358	7.37241934	3.608E-11	9.6604E-09	1.4704064	0.55621495	up	no
ENSG00000168679	SLC16A4	9122	4.39956617	2.87092746	3.73967161	1.28598704	1.57066192	1.75140297	3.919E-11	1.0407E-08	2.38933185	1.25660724	up	yes
ENSG00000159388	BTG2	7832	1.46825505	1.86893937	1.7981576	3.79482941	3.64251023	3.45197614	5.1677E-11	1.3612E-08	0.47159547	-1.0843	down	yes

												7823		
ENSG00000112769	LAMA4	3910	25.3154347	22.7777304	23.2118643	16.0486155	18.2254741	17.0057	6.2935E-11	1.6312E-08	1.3905094	0.4756135	up	no
ENSG00000088826	SMOX	54498	14.245058	8.95304944	11.9706702	6.87904702	5.61385428	5.7424203	6.2916E-11	1.6312E-08	1.92860748	0.94755955	up	yes
ENSG00000156535	CD109	135228	36.7457128	41.9542103	37.504656	52.4927402	52.6957475	56.2594251	6.9582E-11	1.7892E-08	0.71976514	-0.47440187	down	no
ENSG00000092621	PHGDH	26227	15.2732424	19.0096396	16.9218336	27.0391737	24.5004233	24.0286019	7.7944E-11	1.9884E-08	0.67759608	-0.56150256	down	no
ENSG00000162645	GBP2	2634	7.30456817	5.19975929	5.87015462	3.21624812	3.67546446	3.73320004	8.5035E-11	2.1523E-08	1.72937724	0.79025261	up	yes
ENSG00000147119	CHST7	56548	11.398289	6.509596	9.886098	4.945013	3.217495	3.89652	1.1028E-10	2.7696E-08	2.3048278	1.20465897	up	yes
ENSG00000175455	CCDC14	64770	6.83371032	6.44395197	6.40783029	8.84787522	10.3371494	9.15367209	1.2515E-10	3.119E-08	0.6946506	-0.5256406	down	no
ENSG00000139289	PHLDA1	22822	44.5339567	31.7533042	36.072792	25.7511925	21.76004	23.588676	1.3682E-10	3.3837E-08	1.5803122	0.6602096	up	yes
ENSG00000166292	TMEM100	55273	13.3916478	6.98601248	10.8226444	4.83965237	5.14350845	4.68088506	1.6711E-10	4.0404E-08	2.12767369	1.08927691	up	yes
ENSG00000116711	PLA2G4A	5321	4.950425	3.558877	4.397514	2.397097	1.635994	1.744824	1.6613E-10	4.0404E-08	2.23381895	1.15951226	up	yes
ENSG00000196139	AKR1C3	8644	4.9972332	3.36324626	3.64891201	2.17707983	1.33605464	1.71411063	1.6471E-10	4.0404E-08	2.29746095	1.20004034	up	yes
ENSG00000108846	ABCC3	8714	1.67800623	1.74386375	1.61483321	1.00607482	0.99391296	0.96711042	1.7433E-10	4.1836E-08	1.6975182	0.76342705	up	yes
ENSG00000168016	TRANK1	9881	5.48953951	4.54290053	4.67063197	7.12246985	7.20224953	7.18552062	1.9488E-10	4.6423E-08	0.68353826	-0.548906	down	no
ENSG00000004399	PLXND1	23129	27.8872641	29.4780338	29.5289956	20.7499862	22.7451061	21.1785381	2.2742E-10	5.378E-08	1.3435815	0.42608384	up	no
ENSG00000099953	MMP11	4320	2.93871943	2.83232607	2.70186662	4.50401465	4.45009408	4.4758912	2.5311E-10	5.9422E-08	0.63089443	-0.66452949	down	yes
ENSG00000118507	AKAP7	9465	4.81229546	4.35431019	4.2484229	2.70827456	1.75716831	2.48187731	2.9138E-10	6.7915E-08	1.93096449	0.94932163	up	yes
ENSG00000126709	IFI6	2537	71.6675246	49.9010414	59.2241829	39.2441271	35.9679912	31.632344	3.1041E-10	7.1834E-08	1.69211155	0.75882468	up	yes
ENSG00000162745	OLFML2B	25903	59.6480572	60.5542903	58.8643773	42.6582687	46.0667439	45.7146399	3.1626E-10	7.2668E-08	1.33194873	0.41353855	up	no
ENSG00000253368	TRNP1	388610,7207,7217,7218	14.5511785	16.6882315	16.1073304	22.2411103	32.4035381	26.6250402	3.2926E-10	7.5121E-08	0.58258794	-0.77945226	down	yes
ENSG00000166963	MAP1A	4130,23173	18.2249639	17.686585	17.719947	22.6703504	26.892356	25.3110003	3.4373E-10	7.7875E-08	0.71629278	-0.48137869	down	no
ENSG00000269711	ENSG00000269711		0	0	0	0	14.071763	0	4.0739E-10	9.1657E-08	2.1319E-05	-15.5174811	down	yes
ENSG00000173546	CSPG4	1464	9.80946	13.862354	13.53878	18.691793	21.543797	20.429159	4.2486E-10	9.4929E-08	0.61338083	-0.70514502	down	yes
ENSG00000156140	ADAMTS3	9508	4.41750378	2.27231795	3.27395243	1.52629042	1.5327096	1.54225703	4.4626E-10	9.9027E-08	2.16544611	1.11466427	up	yes
ENSG00000065833	ME1	4199,57717	24.334219	26.182657	24.322132	18.675474	17.462675	18.192055	4.7472E-10	1.0463E-07	1.37748439	0.46203597	up	no

ENSG00000159167	STC1	6781	33.9830092	25.4328707	29.6421719	20.3051268	13.5270903	18.2022794	5.0191E-10	1.0987E-07	1.71151943	0.77527767	up	yes
ENSG00000228594	FNDC10	643988	1.219607	1.035271	1.000404	2.698091	2.870472	2.446078	5.6916E-10	1.2376E-07	0.40616691	-1.29985537	down	yes
ENSG00000132846	ZBED3	84327	2.37948817	2.35513464	2.31751261	3.47420773	4.24185102	3.80786273	5.9161E-10	1.2768E-07	0.61195622	-0.70849965	down	yes
ENSG00000120594	PLXDC2	84898	21.4255319	14.3731115	17.017015	11.2510784	10.9256281	11.2458068	5.9508E-10	1.2768E-07	1.58024197	0.66014548	up	yes
ENSG00000130766	SESN2	83667	8.655149	12.387753	9.935103	17.155003	16.683147	16.967152	5.995E-10	1.2778E-07	0.60973961	-0.71373483	down	yes
ENSG00000075223	SEMA3C	10512	24.6626818	23.237878	22.201794	17.9432265	15.4623249	17.1010919	6.1223E-10	1.2964E-07	1.38798283	0.47298972	up	no
ENSG00000134333	LDHA	3939	123.092729	117.180174	116.388548	95.3447338	85.1532438	91.5058837	6.9998E-10	1.4726E-07	1.31123672	0.39092816	up	no
ENSG00000145390	USP53	54532	5.5118512	7.98031453	6.81319512	10.1633637	11.3256585	11.1746883	7.2721E-10	1.52E-07	0.62164894	-0.68582802	down	yes
ENSG00000168000	BSCL2	26580	4.37236018	3.58161649	4.02477084	2.0634517	1.96949137	2.73283445	8.7539E-10	1.818E-07	1.7704909	0.82414943	up	yes
ENSG00000117298	ECE1	1889	30.1387685	28.2450483	28.3666109	20.5787958	22.7784824	21.536112	8.8722E-10	1.8308E-07	1.33681454	0.41879933	up	no
ENSG00000145362	ANK2	287	0.45060943	0.46101415	0.40301074	0.64476589	0.70578187	0.64206122	9.1056E-10	1.8671E-07	0.65975529	-0.59999708	down	yes
ENSG00000151090	THRB	7068	3.28362704	3.09051451	3.02587272	2.28797508	1.85333875	1.87666075	9.2758E-10	1.8901E-07	1.5619897	0.64338494	up	yes
ENSG00000188641	DPYD	1806	8.95943086	8.32607461	7.65273228	4.77630782	5.61348414	5.90292171	9.545E-10	1.9327E-07	1.53063745	0.61413261	up	yes
ENSG00000156804	FBXO32	114907	27.0454346	26.52417	26.2919618	20.48132	19.391093	20.9152146	9.8468E-10	1.9815E-07	1.31377995	0.39372365	up	no
ENSG00000187479	C11orf96	387763	8.245682	5.592788	10.728172	3.643805	2.316556	3.174817	1.0515E-09	2.1029E-07	2.68923517	1.42719592	up	yes
ENSG00000111897	SERINC1	57515	70.732155	72.258003	71.174576	55.162766	55.504044	55.523129	1.0638E-09	2.1144E-07	1.28867448	0.36588789	up	no
ENSG00000272779	BMS1P20	96610	3.189718	4.046037	3.331377	1.309475	0.748244	1.1399	1.0741E-09	2.1218E-07	3.30468764	1.72451392	up	yes
ENSG00000074695	LMAN1	3998	43.5478919	42.3608861	42.855296	34.0517311	32.3366419	33.5100337	1.2299E-09	2.4149E-07	1.28895023	0.36619656	up	no
ENSG00000105664	COMP	1311	2.14504617	1.94343213	2.63418888	0.80046655	0.88083343	1.07577156	1.3391E-09	2.6136E-07	2.43833614	1.28589702	up	yes
ENSG00000162595	DIRAS3	9077	7.03895557	3.48954925	4.78587943	2.04474833	1.40966746	2.35214212	1.3664E-09	2.6508E-07	2.637429	1.39913226	up	yes
ENSG00000153071	DAB2	1601	19.4417644	24.23906	20.4323729	30.3463124	31.0396087	29.8809188	1.4571E-09	2.81E-07	0.70248074	-0.50946942	down	no
ENSG00000213694	S1PR3	1903	4.40618517	4.95553541	4.75738183	3.57096197	2.93075506	3.06448163	1.4832E-09	2.8433E-07	1.47593657	0.56163072	up	no
ENSG00000141068	KSR1	8844	2.10628338	1.24889638	1.53157091	0.92259093	0.70525608	0.74084358	1.5021E-09	2.8627E-07	2.06305994	1.04478573	up	yes
ENSG00000104635	SLC39A14	23516	26.8740224	31.2660297	27.2641412	21.8263904	18.300179	20.1278719	1.5972E-09	3.026E-07	1.4173925	0.50323932	up	no
ENSG00000144711	IQSEC1	9922	3.25079354	3.3400364	3.34502882	4.59820767	4.70609761	4.74410769	1.7239E-09	3.2472E-07	0.70725845	-0.49969059	down	no
ENSG00000135047	CTSL	1514	40.1406062	32.1018003	37.3244062	27.6358753	20.9471069	23.8695089	1.7819E-09	3.3371E-07	1.51225736	0.59670368	up	yes

ENSG00000117525	F3	2152,1272	18.1797662	25.1761784	21.0017565	14.8842869	11.9717293	12.7098531	1.8168E-09	3.3827E-07	1.62659641	0.70185634	up	yes
ENSG00000205413	SAMD9	54809	7.29023155	6.98054477	6.14593777	4.71893172	4.74267873	4.59912664	2.0545E-09	3.8035E-07	1.45203725	0.53807847	up	no
ENSG00000055163	CYFIP2	26999	0.42810178	0.35616058	0.36879223	1.02829092	0.65881261	0.93178702	2.1734E-09	3.9782E-07	0.44028361	-1.18349495	down	yes
ENSG00000144802	NFKBIZ	64332	3.14966364	3.36862921	2.67978323	1.45985464	1.99613832	1.3803887	2.1678E-09	3.9782E-07	1.90185075	0.92740403	up	yes
ENSG00000187193	MT1X	4501	7.45519422	6.12543706	7.33245057	4.23933951	2.95156958	2.74761723	2.3345E-09	4.2491E-07	2.10424374	1.07330183	up	yes
ENSG00000168610	STAT3	6774	20.1840047	16.797628	17.9427355	13.1306007	13.0087497	13.7246017	2.36E-09	4.2715E-07	1.37779536	0.46236163	up	no
ENSG00000120129	DUSP1	1843,11266	62.391224	45.72916	57.225708	35.411415	37.313267	36.627022	2.5312E-09	4.5559E-07	1.51205775	0.59651325	up	yes
ENSG00000059377	TBXAS1	6916	1.44974846	1.65652208	1.48533218	0.74958446	0.70397854	0.86886873	2.7815E-09	4.9787E-07	1.97706682	0.98336163	up	yes
ENSG00000172667	ZMAT3	64393	12.8119093	13.7479044	13.105231	17.7980535	16.7957138	18.0857948	2.8751E-09	5.118E-07	0.7529494	-0.40937518	down	no
ENSG00000115902	SLC1A4	6509	4.58610044	6.46470785	5.2978695	8.74555029	8.78715626	8.44086898	2.9491E-09	5.221E-07	0.62943501	-0.66787067	down	yes
ENSG00000115084	SLC35F5	80255	5.33048492	6.6199294	5.459663	8.10022281	8.46237808	8.93434404	3.1329E-09	5.5164E-07	0.68282994	-0.55040178	down	no
ENSG00000178988	MRFAP1L1	114932	24.8114803	23.8173585	24.4465723	18.2409811	17.7703005	16.3307914	3.3537E-09	5.8731E-07	1.39611228	0.48141498	up	no
ENSG00000139292	LGR5	8549	0.5714494	0.75534676	0.64556032	1.32129875	1.53012783	1.44444303	3.7681E-09	6.5634E-07	0.45912857	-1.12302988	down	yes
ENSG00000145050	MANF	7873	12.1930339	10.4549713	10.6650567	7.1724096	6.96247389	7.20934063	3.8231E-09	6.6236E-07	1.560753	0.64224224	up	yes
ENSG00000111801	BTN3A3	10384	5.22745176	4.1855459	4.59238357	2.85216171	3.03781854	2.94467586	3.9136E-09	6.7443E-07	1.58527746	0.66473537	up	yes
ENSG00000060982	BCAT1	586	13.8644377	18.0784169	14.9943156	21.9877012	22.8569464	23.166057	4.092E-09	7.0144E-07	0.69014386	-0.53503097	down	no
ENSG00000105778	AVL9	23080	2.75075468	3.00816814	2.84295907	4.01214423	4.13261642	3.86172742	4.4049E-09	7.511E-07	0.71643613	-0.48108999	down	no
ENSG00000117862	TXNDC12	51060	11.0265664	12.0098454	11.436758	7.2266429	8.37414242	7.27506518	4.4445E-09	7.5388E-07	1.50696778	0.59164858	up	yes
ENSG00000183671	CMKLR2	2825	10.0782715	11.3499581	10.2645821	17.8377815	14.5910482	15.45286	4.6902E-09	7.9142E-07	0.66189835	-0.59531841	down	yes
ENSG00000101350	KIF3B	9371	13.733909	13.990915	12.648264	19.912094	17.388647	18.164557	5.0341E-09	8.4505E-07	0.72789815	-0.45819149	down	no
ENSG00000157240	FZD1	8321	27.313297	27.874981	26.750149	20.691978	13.770279	18.355978	5.1205E-09	8.5513E-07	1.55132838	0.6335041	up	yes
ENSG00000157191	NECAP2	55707	8.57869489	7.94454235	8.56407348	6.19145012	5.4359521	5.88459897	6.0638E-09	1.0075E-06	1.43257818	0.51861387	up	no
ENSG00000198682	PAPSS2	9060	35.4059859	36.2891666	35.3262124	26.2838544	27.9253143	27.6905422	6.106E-09	1.0093E-06	1.30673678	0.38596857	up	no
ENSG00000176533	GNG7	2788	0.58806133	0.62108421	0.63392884	1.38170234	1.27897199	1.34289357	6.2966E-09	1.0355E-06	0.46035797	-1.11917198	down	yes
ENSG0000	IFITM3	10410	177.75	159.54	171.03	119.39	135.08	127.53	6.7315	1.1015	1.3306	0.4121	up	no

0142089			7153	2669	0607	422	2547	5385	E-09	E-06	656	4806		

Note: A: The control group of SCAPs transfected with shNC Lentivirus, _1 represents the first duplicate, _2 represents the second duplicate, _3 represents the third duplicate; B: The experimental group of SCAPs transfected with shOPTN Lentivirus.

Table S3. The sequencing results of the F1 generation *Optn*$^{+/-}$ mice constructed using CRISPR/Cas9 technology.

**DNA sequence**	5’GNNNNNNNNNGNCNNNATNANTNNATGGCNATCGTTAACTTTGTAATCA<br>ATGAATGATAGGACCTCCCTCAGTCTCTTAGATTTCTAGTCATTAGCAGGAC<br>TTTATCTCTCAATTGGTTTTTATTTGGGAGAATTTGTTGTTTTGAGACACTGT<br>CTCATATAGTCCCACTTGGTAAACTAATTTTATTAGGGCTACTTATGGGGCAG<br>AATCAGTGAATGGTTACTTACAGAAGCAGAAATGACTCAGACAGATGCATC<br>ACCAAGGCCCACCCCTGCCGCAGACCCTCTGGTCCCCTGTGCCTGCGTAGG<br>AAAGAGTCTCTAGAGTAGATGGGCAGAGATTAGGATGTAGTGACAGTCAGA<br>CACACGAGAGTGGTGTTGGATCTGAATGTAATGTTCTGGTTGAGCTTCAGA<br>CTTATATTACAACGAATTATCAGAGGATACAAATCACAAAAAGACAAGATAC<br>ACTGAAATTCACCAGTTACAGCAGAAAGGAATTTGCAGGGACTAATTAAAT<br>GTTTACATTAGGGATAACAAGCCCTGCCTAGGATCAGCCTAAAAGCATCAG<br>GGGGTTGTTAACTCTTGATAGGCCTTAAGAGTAGTGTGTTATCAGGAGCTCT<br>AAATTCTTAGGTCTAGTAAAACTTATCCTGTCTGGAGAGTTCCCCCTTATCA<br>GGGTAGTATATCAACTTATACTTGACATGGAATGTAGCCTGTAGTAAAAGAT<br>TTCTATCTCAGTGAGACTTTTAGTCTCTATCTACAGACAGCTGAGTAAATGA<br>CAGATGTTTTATTGTTTTATGATACAGCTTAATTAATTACTGAATAGGTTAAG<br>CTCCTTGCTGCCTATCTGGAA 3’
